# Design, construction and validation of a magnetic particle imaging (MPI) system for human brain imaging

**DOI:** 10.1088/1361-6560/ad9db0

**Published:** 2025-01-06

**Authors:** Eli Mattingly, Monika Śliwiak, Erica Mason, Jorge Chacon-Caldera, Alex Barksdale, Frauke H Niebel, Konstantin Herb, Matthias Graeser, Lawrence L Wald

**Affiliations:** 1 Dept. of Radiology, A.A. Martinos Center for Biomedical Imaging, Massachusetts General Hospital, Charlestown, MA, United States of America; 2 Harvard-MIT Division of Health Sciences & Technology, Cambridge, MA, United States of America; 3 Department of Electrical Engineering & Computer Science, Massachusetts Institute of Technology, Cambridge, MA, United States of America; 4 Harvard Medical School, Boston, MA, United States of America; 5 Department of Physics, ETH Zurich, Zurich, Switzerland; 6 Fraunhofer Research Institution for Individualized and Cell-Based Medical Engineering, Fraunhofer IMTE, Lübeck, Germany; 7 Institute of Medical Engineering, University of Lübeck, Lübeck, Germany

**Keywords:** magnetic particle imaging, mpi, superparamagnetic iron oxide nanoparticles, cerebral blood volume, functional neuroimaging, SPION, brain imaging

## Abstract

*Objective.* Magnetic particle imaging (MPI) was introduced in 2005 as a promising, tracer-based medical imaging modality with the potential for high sensitivity and spatial resolution. Since then, numerous preclinical devices have been built but only a few human-scale devices, none of which targeted functional neuroimaging. In this work, we probe the challenges of scaling the technology to meet the needs of human functional neuroimaging with sufficient sensitivity for detecting the hemodynamic changes following brain activation with a spatio-temporal resolution comparable to current functional magnetic resonance imaging approaches. *Approach.* We built a human brain-scale MPI system using a mechanically-rotated, permanent-magnet-based field-free line (FFL) ($1.1\,\mathrm{Tm}^{-1}$) with a water-cooled, 26 kHz drive coil producing a field of up to 7 mT$ _{\mathrm{peak}}$, and receive coil that can fit over a human head. Images are acquired continuously at a temporal resolution of 5 s/image, controlled by in-house LabView-based acquisition software with online reconstruction. We used a dilution series to quantify the detection limit, a series of parallel-line phantoms to assess the spatial resolution, and a large ‘G’ shaped phantom to demonstrate the human-scale field of view (FOV). *Main results.* The imager has a sensitivity of about 1 *µ*g$ _{\mathrm{Fe}}$ over a 2D imaging FOV of 181 mm diameter(132 pixels) in a 5 s image. Depending on the image reconstruction used, the spatial resolution defined by 50% contrast between adjacent lines was 5–7 mm. *Significance.* This proof-of-concept system demonstrates a pathway for human MPI functional neuroimaging with the potential for an order of magnitude increase of sensitivity compared to the other human hemodynamic imaging methods. It demonstrates the successful transition of the FFL based MPI architecture from the rodent to human scale and identifies areas which could benefit from further work.

## 1. Introduction

There are various *in vivo* neuroimaging modalities for applications ranging from anatomical structure studies to measurements of functional tissue properties. Each technique has a unique combination of biological contrast specificity, tissue-penetration capability, signal sensitivity, spatiotemporal resolution, as well as its injected agents’ shelf-life and toxicity profile. magnetic particle imaging (MPI) was introduced in 2005 as a noninvasive imaging modality to map the distribution of injected superParamagnetic iron oxide nanoparticles (SPIONs) by exploiting their nonlinear response to an applied oscillating magnetic field. Localization is achieved by superimposing gradient fields that result in signals only arising from a small field-free region that is swept over the imaging space (Gleich and Weizenecker 2005). MRI detects nuclear spin magnetism directly and injected magnetic contrast agents such as gadolinium compounds or SPIONs only indirectly through their relaxation effect on the surrounding water’s nuclear spin resonance. Unlike MRI, MPI detects the SPION’s magnetization directly. MPI and MRI share the use of applied magnetic fields and the detection of time-varying magnetic moments through Faraday induction. However, as a tracer detection method, it is more appropriately classified with other tracer mapping modalities like single photon emission computed tomography and positron emission tomography.

The SPION detected by MPI will not cross the healthy blood-brain barrier, and therefore an MPI brain image reflects the cerebral blood volume (CBV). This makes it an attractive technology for imaging the cerebral vasculature, as well as blood volume, flow and perfusion (Wu *et al* 2019, Mason *et al* 2023). The signal level is proportional to the CBV and is unobscured by non-blood pool signals which contribute to CT or MR images. The long circulation times of coated SPIONs (Khandhar *et al* 2017) provides the ability to regionally monitor the CBV for changes. MPI has been used in this way to detect murine gut bleeds (Yu *et al* 2017), perfusion in murine stroke (Ludewig *et al* 2017), intracranial hemorrhage (Szwargulski *et al* 2020), and traumatic brain injury in preclinical studies (Orendorff *et al* 2017). More recently, MPI has been assessed for mapping CBV changes following hypercapnia in rats (Mason *et al* 2023).

MPI detects and spatially localizes the magnetization changes in an injected SPION tracer in response to an applied ‘drive’ field, which is typically (though not necessarily (Tay *et al* 2019a)) harmonically pure. Since the SPION magnetization response to the applied drive field is nonlinear (modeled by the Langevin function in the relaxation-free case (Shliomis 1972, Kluth 2018)), the magnetization signal detected by the receive coil via Faraday induction contains harmonics of the drive waveform. Application of a field gradient, either in the form of a field-free-point or field-free line (FFL) (Weizenecker *et al* 2008, Knopp *et al* 2010a, Greiner *et al* 2022) saturates the particles outside of the zero-crossing region, reducing their Faraday response and thereby localizing the signal to the field-free region. The field-free region is then swept in space to provide the map of SPION distribution, which can be reconstructed with either a forward model inversion of a measured (Weizenecker *et al* 2009) or simulated linear model of the signal (Rahmer *et al* 2009, Knopp *et al* 2010b), or by mapping the signal to the field-free region’s trajectory in space (*x*-space MPI) (Goodwill and Conolly 2010, 2011).

For successful functional neuroimaging of the CBV response to brain activation in humans, the device should be capable of imaging a human head with 6 mm spatial resolution every 5 s over the course of 30 min. This minimum spatial resolution reflects that used in many useful functional magnetic resonance imaging (fMRI) studies which typically smooth to a 4–12 mm spatial resolution (Huettel *et al* 2014). Other fMRI studies have also well-characterized the timecourse of CBV changes (Mandeville *et al* 1998, Mason *et al* 2023), and show that the hemodynamic response reaches 90% of its peak in about 5 s (DeYoe *et al* 1994), therefore this or better temporal resolution is needed to capture the dynamic changes. The functional MPI scanner needs to be able to stably image for at least 30 min to perform studies similar to a typical fMRI study. While these specifications offer no advantage over fMRI, which also offers a wealth of useful anatomical and diffusion contrasts, theoretical analysis (Mason *et al* 2017) and preliminary fMPI studies in rats (Mason *et al* 2023) suggest that the sensitivity of fMPI can potentially exceed that of fMRI, due to its detection of the much larger SPION magnetization (compared to the hydrogen nuclear magnetic moment) and the lack of confounding signals from the non-blood pools (which contribute physiological noise to fMRI (Triantafyllou *et al* 2006, Krüger *et al* 2010)).

Although no human MPI has been performed, there are currently a few systems under development for human-scale MPI. Each has been tailored for a separate application and thus would be poorly suited for fMPI. Examples include a device tailored for the imaging of stroke perfusion deficits in a portable setting which employed low power and achieved portability at a cost of spatial resolution (Graeser *et al* 2019, Thieben *et al* 2024). Others have built systems for interventional radiology of limbs, an application which does not require high sensitivity or long-term stability (Vogel *et al* 2023). Other systems have been presented at early stages of development (Top *et al* 2017, Mason *et al* 2024, Nomura *et al* 2024, Yoshida *et al* 2024) but very little experience exists with human-scale MPI scanner hardware, especially for functional neuroimaging applications.

In this work, we introduce a mechanically rotating FFL-based Magnetic Particle Imager designed for human brain imaging. We describe the design of each subsystem, including the overall mechanical gantry which rotates the permanent-magnet-generated FFL and high-power electromagnet shift coils, a 26.3 kHz drive coil which generates the SPION response and the drive chain filter, as well as the receive coil and low noise electronics. We characterize its spatial resolution, sensitivity, field of view (FOV) and temporal stability in phantom imaging, including phantoms at the human brain scale.

## 2. Methods

### 2.1. MPI scanner and acquisition scheme

#### 2.1.1. Architecture of the human scale MPI scanner

**Figure 1.**
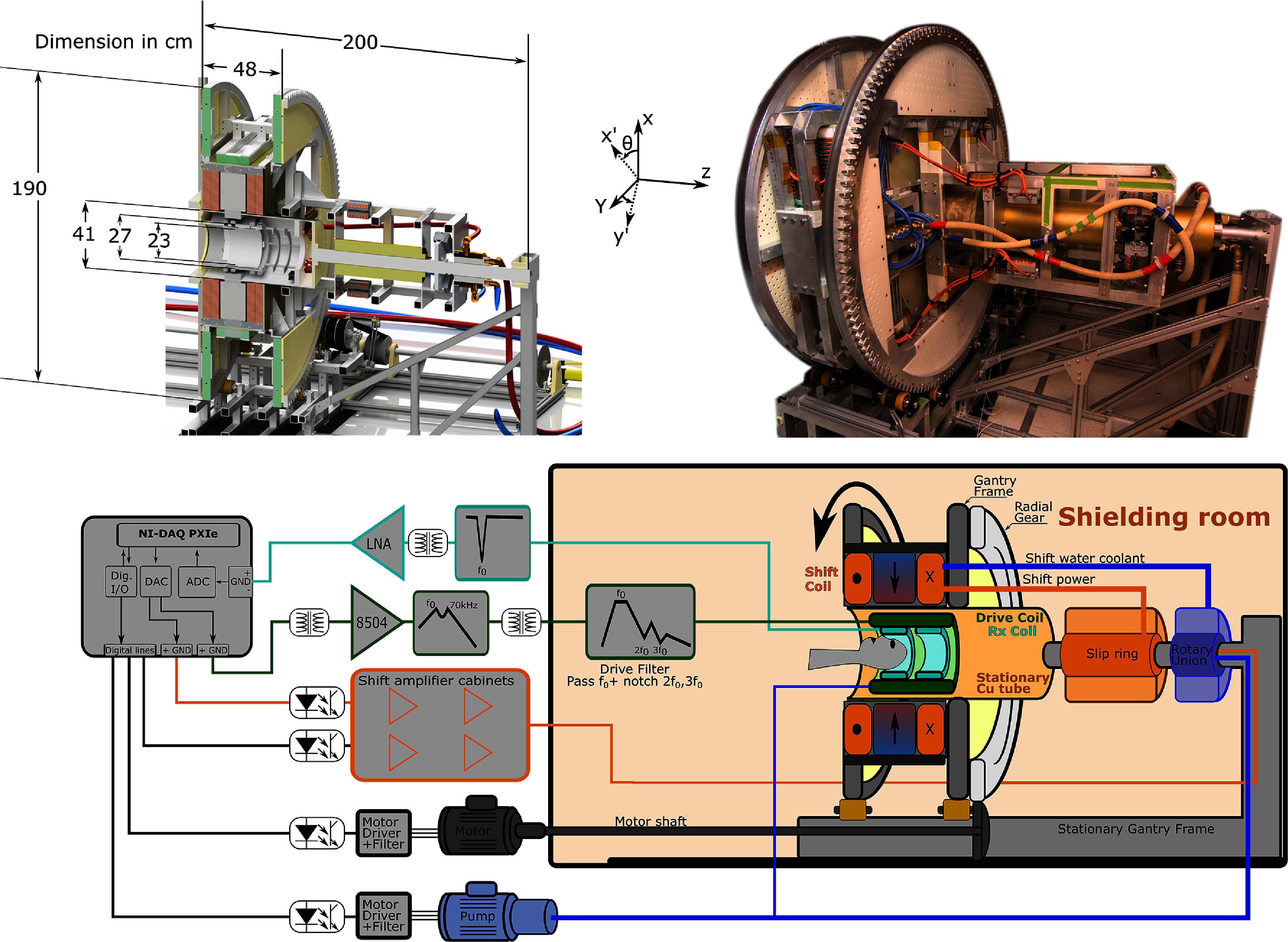
Top left: Digital rendering of a cross-section of the imager with major dimensions indicated. The drive coil’s inner diameter is 27 cm and the major and minor axes of the elliptical receive coil are 23 cm and 16 cm (not shown). Top right: photograph of the imager from a similar angle. Bottom: Schematic of the main subsystems and an illustration of the system. The primed coordinated directions (i.e. *x*’, *y*’) indicate the rotating coordinate frame, where $\mathrm{\theta}$ is the gantry rotation angle.

The system architecture refines our previously presented rodent-scale imager (Mattingly *et al* 2022a) and scales it to a geometry suitable for human head imaging. A preliminary version of the scanner has been presented in abstract form (Mattingly *et al* 2024b). The scanner is based on a FFL architecture (Weizenecker *et al* 2008), with the FFL gradient generated by a pair of mechanically rotated NdFeB permanent magnets. An electromagnetic coil pair shifts the zero-crossing of the FFL’s gradient across the human head ($\mathrm{\pm~10\,cm}$) to form a projection at each rotation angle. A single drive coil produces an oscillating field at 26.3 kHz to drive the SPIONs in and out of (partial) saturation. The d*M*/d*t* response is recorded as the EMF induced in a single first-order gradiometer receive coil. In this receive coil, the head occupies the windings of one of the counterwound pair. Figure 1 shows an illustration of a head in the coil, a system schematic, a cross-sectional drawing, and a scanner photograph.

##### 2.1.1.1. Mechanical rotating gantry:

**Figure 2.**
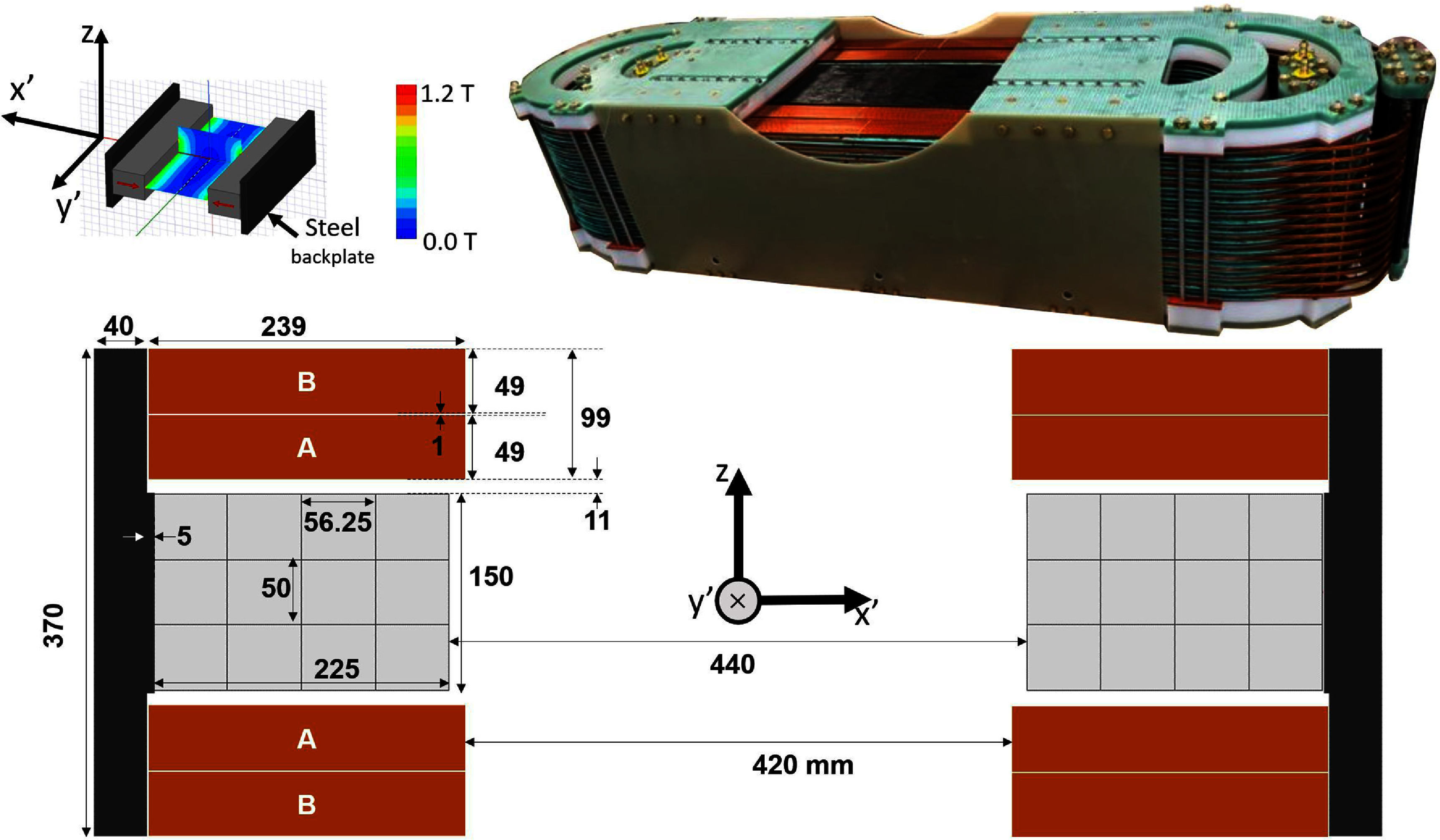
Top left: Simulation of the permanent magnets showing the location of the FFL. Top right: Photograph of the fully constructed shift coil (background removed for clarity). Bottom: Dimensions of the shift coils and FFL magnets. The primed coordinated directions (i.e. *x*’, *y*’) indicate the rotating gantry coordinate frame, where the axis of rotation is *z*.

The rotating gantry serves to move the FFL through the radial projection angles needed for the 2D image. The roughly 1500 kg circular rotating assembly includes the FFL’s permanent magnets and the shift coils. The assembly rotates on the two 1.9 m diameter outer rings formed from 50.8 mm square solid aluminum bars. These were rolled into semicircles, and assembled with dielectric breaks along the *x*’–*z* plane (the primed coordinate directions are the rotating coordinate frame as in figure 2) to reduce eddy currents from the time-varying shift fields. They roll on 8 polyurethane rollers supported by a similarly welded aluminum stand. A frame extension on the service end of the scanner (+*z* end) supports the rotating portion of the electrical slip rings (MT080-P16300-1KV, Moflon Technology, Shenzhen, China) and cooling water’s rotary union (MEPH200-09-ID80, Moflon Technology, Shenzhen, China) which allows delivery of current and water to the rotating shift coils. This allows unlimited rotation without having to reverse the gantry to unwrap cables and water lines. This is essential because of the large inertia of the system and 5 s temporal resolution target. A fully electrically rotated FFL is possible (Weizenecker *et al* 2008), but would require considerably more electrical complexity and power requirements. Optimizing the electromagnetic design can temper the power requirements, though not entirely (Erbe *et al* 2011). Alternatively, a rotating Halbach array could have been used for the generation and rotation (Weber *et al* 2018, Ergor and Bingolbali 2022), though these designs are more mechanically complex and may be challenging to adapt to this large scale. Single-sided MPI systems have also been suggested for human imaging, but lack the depth penetration and scan volumes necessary (Sattel *et al* 2009, Pagan *et al* 2021), and are generally more well-suited for specialized applications such as breast cancer imaging.

The rotation is powered by a 3 HP electric motor coupled to the gantry by a shaft passing through the wall of the shielded room (which houses the scanner). The shaft is coupled to a timing belt and pinion engaging with a large (1.9 m diameter) circumferential gear bolted to the gantry ring. With the motor at its operating speed of 580 RPM, the 1:10 worm gear on the motor together with the 16:155 gearing on the wheel rotates the gantry at 6 RPM (one rotation every 10 s) allowing an image time-series to be acquired with a temporal resolution of 5 s (an image every 180^∘^). The gantry angular position is tracked with an angular encoder as well as an optical homing switch. The homing switch allows for images to be triggered at consistent angular positions, and therefore prevents angle offset errors from adding image instability.

##### 2.1.1.2. FFL magnets:

A gradient above 1 T m^−1^ is needed to achieve the target spatial resolution of 5–6 mm given the magnetization curve of the commonly used, high performance SPIONs such as Synomag-D (Micromod Partikeltechnologie GmbH, Rostock, Germany), without deconvolving the point-spread function, which comes at the cost of sensitivity as the deconvolution operation is ill-posed. Synomag-D particles have a full-width half maximum magnetization response of about 6 mT for the third harmonic component of the signal as measured with our in-house magnetic particle spectrometer using the ‘system matrix mode’. This mode of operation records the amplitude of the harmonic signal with a slowly varying (11 Hz) external bias field (Mattingly *et al* 2024a). This would be the through-plane kernel (i.e. kernel in *z*), the MPS is not currently configured to measure the in-plane (*x*’–*y*’) kernel. The FFL magnets, a custom assembly by BJA Magnetics, Leominister MA, was formed from two 850 $\mathrm{mm}$ long NdFeB rare-earth permanent magnets arranged in opposition and attached to a steel back-plate. Figure 2 shows the configuration of these magnets as well as the dimensions of the assemblies. The FFL magnets consist of 204 (17 × 3 × 4 in *y*’,*z*, and *x*’ respectively) NdFeB blocks each measuring 50 × 50 × 56.25 $\mathrm{mm^3}$(magnetized along the 56.25 mm dimension), resulting in an assembly that is 850 × 150 × 225 $\mathrm{mm^3}$. Each of the constituent blocks are coated in an insulating epoxy to prevent the induction of large eddy currents by the time-varying shift fields. The steel back plate reduces the reluctance of the effective magnetic circuit thereby increasing the gradient strength as well as partially containing the fringing fields. The back plate is formed from laminated M6 transformer steel to reduce eddy currents. Both the field and eddy current heating were simulated in Ansys Maxwell (Ansys Inc. Canonsburg, PA, USA). Further discussions on the magnet designs are found in reference (Mason 2020). The gradient achieved in the constructed magnet was characterized using a 3-axis Hall magnetometer (Metrolab Technology SA, Geneva, Switzerland, model Metrolab THM 1178) and a robotic positioner. The measured FFL gradient strength in *z* is 0.85 T m^−1^ and 1.13 T m^−1^ in *x*’.

##### 2.1.1.3. Shift coils and amplifiers:

The shift coils produce a roughly uniform field which ramps slowly (2.7 Hz rounded triangular waveform) to sweep the zero-crossing of the FFL gradient across the imaging volume, generating a 1D projection at each rotation angle. The shift coil is composed of 4 coils; an inner and outer ‘racetrack’ coil (labeled A and B in figure 2, respectively) on each side of the head. Each racetrack coil has 7 turns per layer and 30 layers total, all built from Kapton-wrapped copper hollow conductor wires (6 × 6 $\mathrm{mm^2}$ square cross-section). Water coolant flows through the hollow-core wire in its 3 mm diameter central axial hole. Breaking the shift coils into an inner and outer coil doubles the number of parallel water circuits. Coolant flow is increased since each circuit’s length is reduced by a factor of two. The configuration also requires lower voltage amplifiers (although 4 of them). The simulated and measured electrical impedances of the shift coil racetrack coils are in table 1. The impedance was measured with an LCR meter at 100 Hz. To determine the mutual inductance, we connected an inner and outer racetrack in series such that their fields aid each other, and measured the total inductance to calculate the mutual inductance ($L_\textrm{total} = L_\textrm{outer}+L_\textrm{inner}+2M$). The expected heat load for the 250 A$ _{\mathrm{peak}}$ triangle wave needed for a FOV of 19 cm is calculated from the measured resistances to be 29.5 kW and needs to be removed by the water system.

**Table 1.** Measured and simulated resistance and inductance of the shift coils. The simulation values were performed using Ansys Maxwell at 7 Hz, and the measurements were done at 100 Hz using the Agilent 4263B LCR Meter.

	Inner racetrack (‘A’)		Outer racetrack (‘B’)	
	Measured	Simulated	Measured	Simulated
Resistance (DC, 25 ^∘^C)	310 m$\Omega$	301 m$\Omega$	399 m$\Omega$	385 m$\Omega$
Inductance	26 mH	29 mH	41 mH	43 mH

	Measured	Simulated		

Mutual inductance (inner and outer)	22.6 mH	25 mH		

Four switch-mode amplifiers (International Electric Company Oy, Helsinki, Finland, model MPS 300–750) power the four shift coils. With a maximum current of 300 A$ _{\mathrm{peak}}$, each amplifier is capable of driving a racetrack coil at the 250 A$ _{\mathrm{peak}}$ and 100% duty cycle needed for continuous 19 cm FOV imaging.

The shift amplifiers are fully isolated from the operating console via optical isolation for both digital interfaces (e.g. enable lines) and analog signals (input and voltage/current monitors) to isolate the acquisition and control electronics from potential noise from these switch-mode amplifiers. The digital signals are isolated with digital optoisolators, and the analog signals are isolated via ISO224 isolation amplifiers (Texas Instruments, Dallas, TX, USA).

##### 2.1.1.4. Bore tube and shielding:

**Figure 3.**
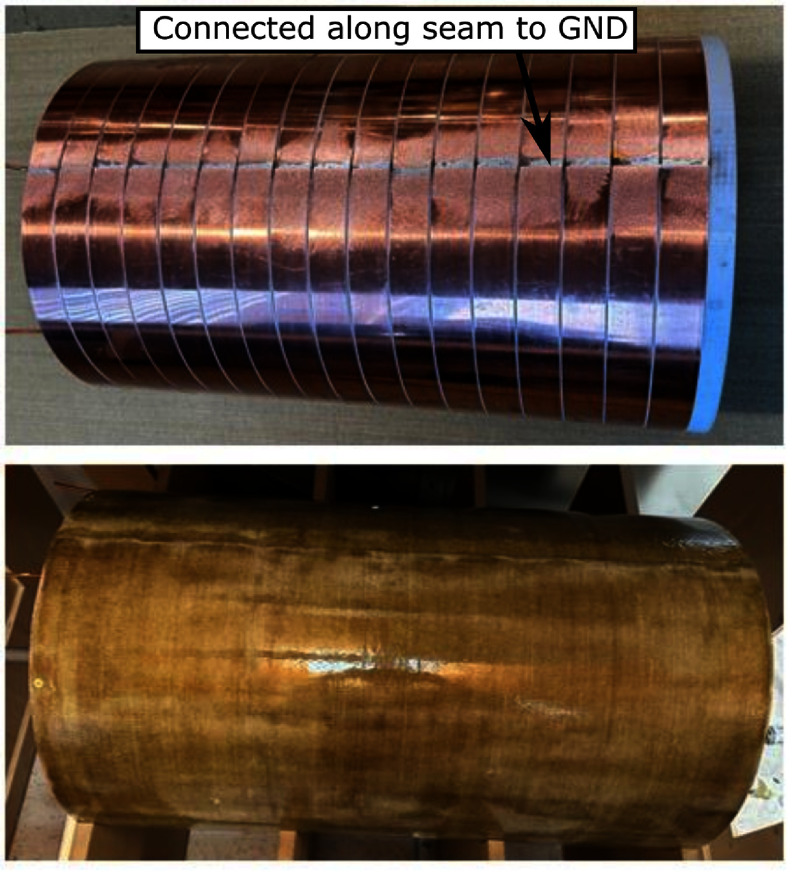
Photograph of the shielded bore before and after the fiberglass wrapping. The rings are electrically connected along a single grounded ‘spine’ formed from a hollow conductor copper wire (4 mm OD, 2 mm ID). Besides that one connection point, the rings are electrically isolated to limit large eddy current loops induced by the shift coils’ d*B*/d*t* while still effectively shielding the receive coils from external fields in the *Z* direction.

Figure 3 shows the 40 cm diameter copper shielding tube used to isolate the sensitive receive electronics from noise generated by external sources such as the shift coils (or elsewhere). The tube is stationary (non-rotating) and is positioned between the rotating shift coils and the drive (and receive) coils. It is constructed from 17 copper rings, each 1 mm thick and 38 mm wide with electrically isolated gaps between them to prevent the induction of eddy currents by the time-varying shift fields. Each ring is electrically connected along a single ‘spine’ to ensure they are all grounded to the same point. Considerable currents circulate in these rings because this shield is strongly coupled to the drive coils. As a point of reference, the FEMM 4.2 simulation show that a single representative 38 mm wide shield loop must support a current as high as 237 A$ _{\mathrm{peak}}$ for a drive current of 50 A$ _{\mathrm{peak}}$ in the drive coil. As the rings are in a strong magnetic field (from the shift, FFL magnets, and drive field) any eddy currents present experience substantial Lorentz forces. They are mechanically stabilized by wrapping them in epoxy-impregnated fiberglass sheets. Without this support, the rings would be free to mechanically deform in a manner coherent with the drive field, introducing a nonlinearity in the drive field that could generate harmonics. These harmonics would be picked up by the receive coils as a background signal.

##### 2.1.1.5. Drive coils, amplifier, and filter:

The drive coil was designed to achieve 7 mT$ _{\mathrm{peak}}$ amplitude when driven with 60 A$ _{\mathrm{peak}}$ of current at 26.3 kHz. A period of exactly 38 ms is used to ensure each period has an integer number of samples and is digitized in an identical manner. The drive coil fits within the 40 cm diameter shielding tube, and has a 27 cm inner diameter to fit the head-sized receive coil.

**Figure 4.**
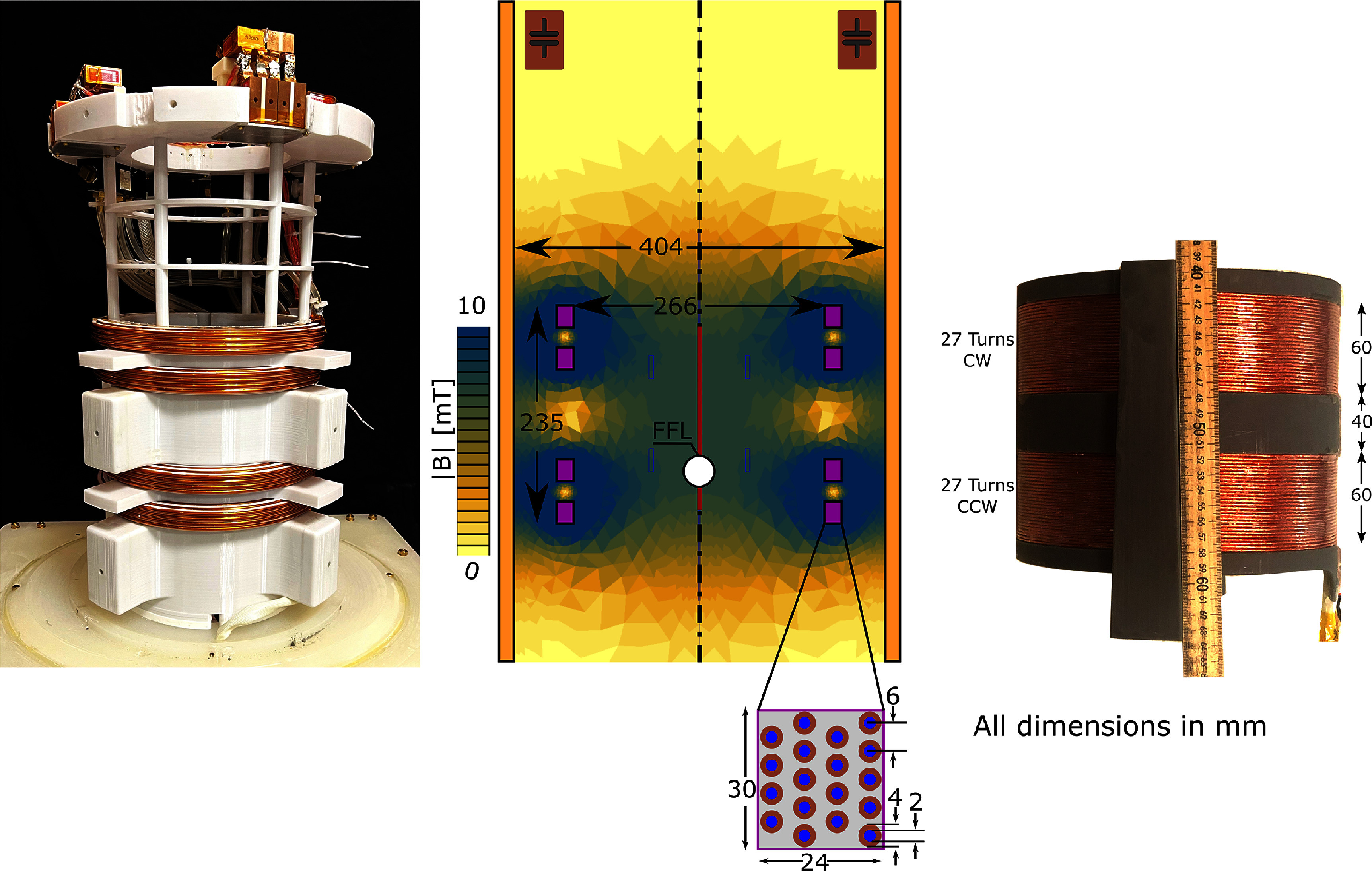
Left: Photograph of the drive coil, shown outside of the bore. Center: FEMM 4.2 axisymmetric simulation of the magnetic fields produced by the drive coil with 60 A$ _{\mathrm{peak}}$ at 26.3 kHz. Right: Photograph of the receive coil with dimensions given in mm. Note: all three are not at the same scale.

The drive coil consists of four individual winding ‘modules,’ each consisting of 18 turns of 4 mm outer diameter hollow copper tubing (2 mm inner diameter for coolant flow) as seen in figure 4. The spacing of these four modules was optimized for homogeneity along the center axis, weighting the optimization function by the receive coil’s sensitivity profile (i.e. the drive field is most uniform where the receive coil is most sensitive). The presence of the 40 cm diameter shielding tube strongly affects the drive coil’s magnetic field. The coils were simulated and optimized with an in-house developed Biot–Savart solver. This solver accounts for induced eddy currents in the shield by modeling the system as a matrix of coupled inductors, then using an equivalent circuit model (Mattingly 2024). The solver was written in the Julia Programming Language^8^8available at github.com/EliMattingly22/Biot.jl., and following these simulations, FEMM 4.2 was used to verify the findings. The total inductance of the drive coil when inside the bore is 643 *µ*H. The inductive reactance of each drive module was nulled with a series capacitor bank totaling 244 nF and composed of 4 CSM Nano capacitors (Celem, Jerusalem, Israel) to distribute the voltage drop across the coil. The capacitors were connected using green-laser-welded copper tabs that are soldered to, to improve the connection linearity and robustness because connection nonlinearity (e.g. from screw connections) can introduce signal instability (Wilkerson 2010, Aderhold *et al* 2023).

Despite its higher electrical resistance than a similar-sized Litz wire bundle, the hollow conductor wire with cooling water was chosen to facilitate cooling. Using analytical models for the viscous resistance of the water flowing within the hollow conductors (Zigrang and Sylvester 1985) at 40 PSI, we calculate the thermal resistance for each of the four modules is 60 mK W^−1^, for a net effective thermal resistance of 15 mK W^−1^. With 50 A$ _{\mathrm{peak}}$ current (typical operating conditions) the drive coils dissipate about 1.6 kW, yielding an expected temperature rise of 25 K.

The drive amplifier is an AE Techron 8504 (AE Techron, Elkhart, IN, USA), which is a 4 kW switch-mode amplifier. Because the amplifier has switching noise at multiples of 125 kHz, we have a low-pass filter with a cutoff frequency of about 70 kHz immediately after the amplifier to shunt those frequencies out of the signal. Following that filter, the power is coupled to the main filter with a ferrite transformer to provide common-mode noise isolation and mitigate grounding issues. The transformer core is a pair of U-shaped N87 ferrite cores (U 141/78/30, TDK Lambda, Tokyo, Japan) which were glued together with a $\sim \mathrm{1\,mm}$ gap between the two halves to form a toroidal shape. The measured magnetizing inductance is 1.55 mH and turns ratio is 1.87:1 (inductance ratio = 1:3.51, where the high-turns side is the filter side). The primary-referred leakage inductance is approximately 320 *µ*H, based on the capacitor which resonated with that leakage inductance. We were not able to measure any increased distortion in the current due to the ferrite transformer using Rogowski coil current monitors (Ramboz 1996, Ferkovic and Ilic 2007) located before and after the transformer.

Following the transformer, the drive filter is similar to the design presented previously (Mattingly *et al* 2022b). The filter is fully balanced, has a T-type band-pass section, then notch sections for the 2nd and 3rd harmonics of the drive frequency, and a capacitive impedance matching section, which couples to the drive coil. The filter schematic is shown in the supplemental data.

##### 2.1.1.6. Receive coil and preamplifier:

The receive coil is a gradiometer where each half is a 27-turn solenoid with an elliptical cross-section (major diameter = 230 mm, minor diameter = 160 mm) wound with type-2 Litz wire with 115 strands, 36 AWG and PFA coating (New England Wire Technologies, Lisbon, NH, USA) . The inductance of the receive coil is 324 *µ*H and resistance is 1.8 $\mathrm ~\Omega$ at 100 kHz measured with an Agilent 4263B LCR Meter.

**Figure 5.**
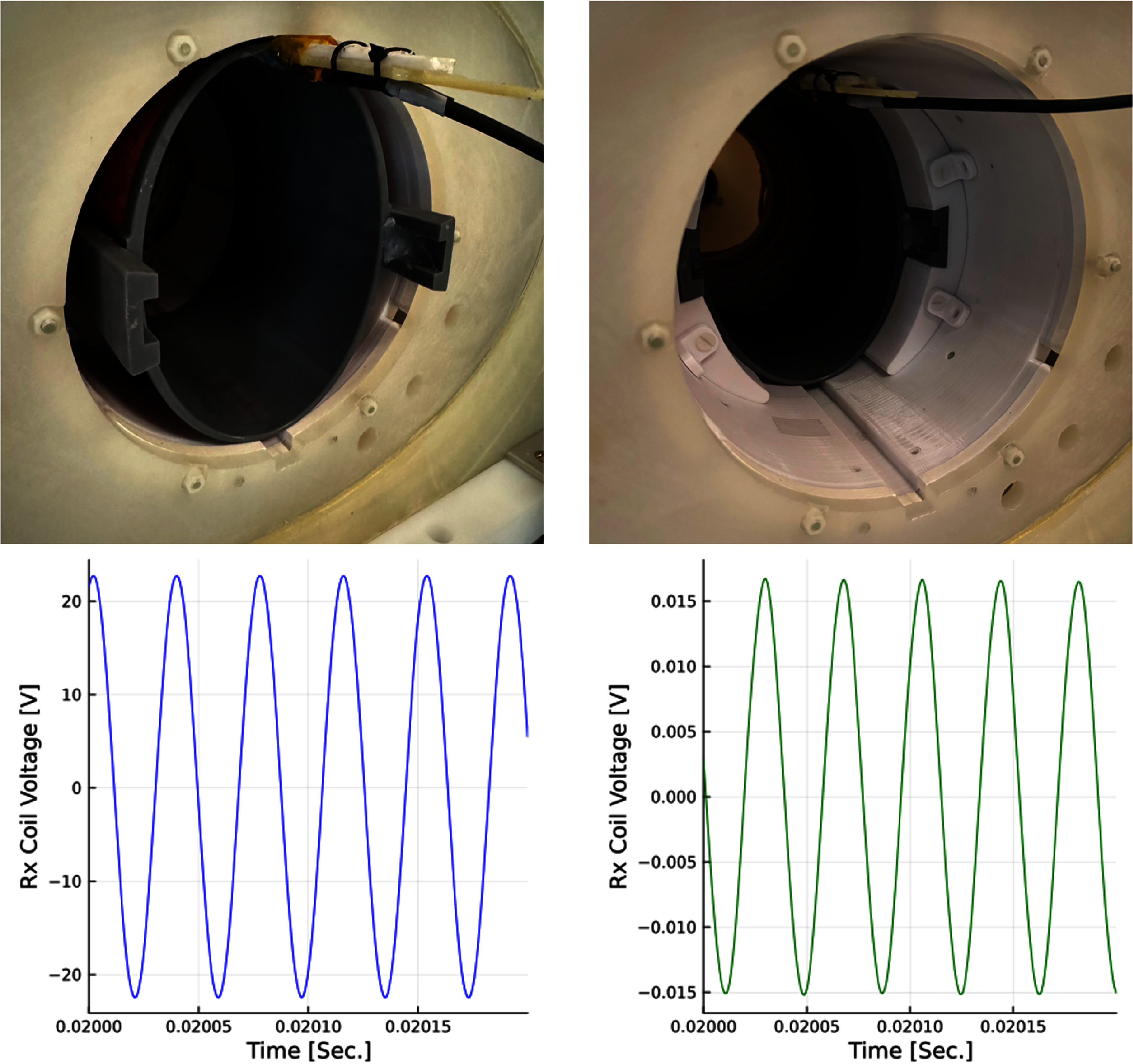
Left Column: Receive coil feedthrough with the receive coil halfway out and drive current at 1.67 A$ _{\mathrm{peak}}$. Right Column: receive coil feedthrough with the receive coil positioned to minimize induced voltage and drive current at 16.4 A$ _{\mathrm{peak}}$.

The receive coil’s gradiometer design reduces unwanted feedthrough of the 26 kHz drive into the receive chain. We measure the feedthrough attenuation of the drive and Rx implementation by first placing the receive coil halfway out of the drive coil to maximize the induced voltage (figure 5, left) and comparing it to the induced voltage with the Rx coil optimally placed. During this process, the drive coil is powered with 1.67 A$ _{\mathrm{peak}}$ and the receive coil voltage is recorded with a floating oscilloscope (RTH1002 Rohde & Schwarz, Munich, Germany). The coil is then positioned to minimize the induced voltage with drive coil current increasing to 16.4 A$ _{\mathrm{peak}}$ (figure 5, right). The feedthrough attenuation is:

\begin{equation*} \mathrm{A_{Grad}} = \mathrm{\frac{\textit{V}_{Rx,Out}}{\textit{V}_{Rx,In}}\cdot \frac{\textit{I}_{Drive,In}}{\textit{I}_{Drive,Out}}}\end{equation*}

where $\mathrm{\textit{V}_{Rx,Out}}$ and $\mathrm{\textit{I}_{Drive,Out}}$ is the receive coil voltage and drive coil current with the receive coil halfway removed. The ‘in’ subscript indicated the coil positioned with the minimum induced voltage. The measured feedthrough attenuation of the gradiometer is 83 dBc, as seen in figure 5. The receive coil indexes in the drive coil with dovetail wedges, so it can easily be removed and replaced with other application-specific coils for increased sensitivity (Graeser *et al* 2020, Chacon-Caldera *et al* 2024). The process of inserting a receive coil and fine-tuning its position for minimum feedthrough typically takes less than one minute.

**Figure 6.**
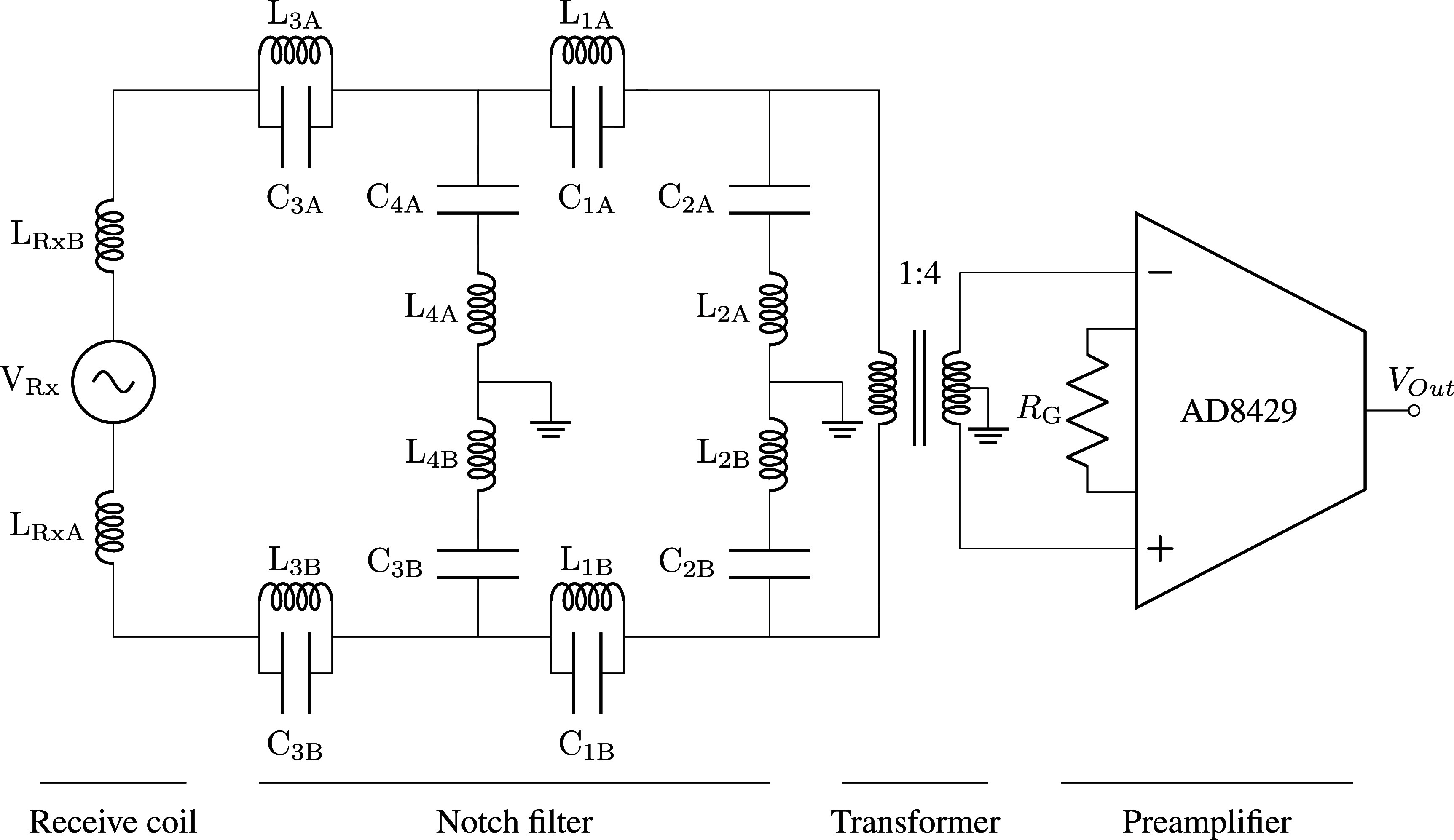
Receive filter and preamp circuit model. The transformer coupling scales the effective impedance of the Rx coil and simultaneously mitigates common-mode noises, and gives a path for bias currents to flow. $\mathrm{C}_{\mathrm{{1/3A/B}}} = 170\,\mathrm{nF}$, $\mathrm{L}_{\mathrm{{1/3A/B}}} = 190\,\mu\mathrm{H}$, $\mathrm{C}_{\mathrm{{2A/B}}} = 9.72\,\mathrm{nF}$, $\mathrm{L}_{\mathrm{{2A/B}}} = 1.88\,\mathrm{mH}$, $\mathrm{C}_{\mathrm{{4A/B}}} = 8.66\,\mathrm{nF}$, $\mathrm{L}_{\mathrm{{4A/B}}} = 2.12$ mH, $\mathrm{R}_{\mathrm{{G}} = 31.1~\Omega}$. The transformer has a turns ratio of 1:2, for an inductance ratio of 1:4. Following the AD8429, there is low-pass filtering (cutoff $\approx250\,\mathrm{kHz}$), and single-ended to differential conversions, which are not shown here for simplicity.

The receive coil is connected to a fully balanced notch filter (Schumacher *et al* 2020), transformer (1:2 turns ratio), and preamplifier as illustrated in figure 6. Having the shunt section inductors in the receive filter (i.e. $\mathrm{L_{2/4}}$) and the magnetizing inductance of the transformer be large is important to prevent the reactance of the receive coil from forming a significant voltage divider with the reactance of these inductors. The secondary of the transformer has a center-tap to ground to give the bias currents a path to flow and not saturate the input. The preamplifier is an AD8429 (Analog Devices, Wilmington, Massachusetts) instrumentation amplifier with a theoretical input noise of 1.2 nV/$\sqrt{\mathrm{Hz}}$ and a gain of 200. Following the first-stage preamplifier, there is a low-pass filter for anti-aliasing (cutoff $\mathrm{\approx~250\,kHz}$), and a single-ended to differential conversion.

##### 2.1.1.7. Data acquisition system, and software:

The imager control software generates the drive and shift waveforms, controls the motor and gantry, and ensures accurate synchronization to the digitization of the preamplified SPION signal. It also records data from other peripherals and monitors such as the gantry rotary position encoder and drive current monitor. The console operates on a single NI PXIe-1073 that has PXIe-6363 and PXIe-6361 X-Series DAQs to digitize the received signal and generate the analog drive and shift waveforms, and the same PXIe modules record digital data from the rotary shaft encoder. It runs on custom-written LabVIEW software (LabVIEW 2021, Emerson Electric Co., St. Louis, MO). The start of the ADC and DAC is triggered off of each other to ensure consistent timing.

Each image is acquired in a 180-degree rotation of the gantry in 5 s. In that time, 27 projections are acquired, so the shift frequency is 2.7 Hz (2 projections per period of the triangle wave). Within each of the projections the data is discretized into 132 equal segments, called ‘read-outs’ (ROs). Each RO contains data from exactly 35 periods of the drive frequency (1.33 ms) and digitized at 1 mS s^−1^. The RO time domain data are Fourier transformed and the complex signal intensity at the 2nd through 9th harmonics is saved and the other frequency components are discarded. The mean shift currents, which are monitored via the integrated current monitor on each amplifier, and the gantry position are also recorded during the 1.33 ms RO. The receive signal components are binned and mapped according the shift amplitude and gantry angle to form the sinogram consisting of the recorded harmonic amplitude as a function of gantry angle and shift field amplitude. The operator is supplied with an online view of each image using a simple inverse Radon transform of the 3rd harmonic data, but the sinograms of the 2nd through 9th harmonics are saved for more advanced offline reconstructions.

### 2.2. Image reconstruction

Two different offline 2D reconstruction algorithms are used: an inverse Radon reconstruction, and a forward-model iterative reconstruction. Both used only the 3rd harmonic (although the higher harmonics are saved). The 3rd harmonic is valid to use for a 2D acquisition of in-plane SPION distributions due to the symmetry of the 3rd harmonic sensitivity profile (Mason *et al* 2022). Each complex projection (column in the 3rd harmonic sinogram) has a linear complex baseline subtracted off of it by taking a linear fit between the mean of the first 2 points and mean of the last 2 points and then subtracting this from the measured data. Essentially, we assume there is no signal at the perimeter of the FOV. The angle for each projection is the mean measured angle of all the ROs in that projection. As there are occasional spurious spikes in the received signal (arising from the shift system), these points are identified in the sinogram domain as points more than three local scaled median absolute deviations away from the local median within a three-element moving window using MATLAB’s built-in function *isoutlier*([data],‘movmedian’,3). While we do not know the root cause of the noise introduced by the shift system, it could result from either insufficient filtering of the current leads or from intermittent connections in the grounds causing small arcs that have not been found.

The forward model-based (iterative) reconstruction improves on the inverse Radon reconstruction by including three main pieces of information: The measured gantry angle at each RO, the measured shift current, and an assumed width of the SPIONs’ point-spread function. The forward model is built with a simulator in MATLAB 2021 (Mathworks, Natick, MA, USA), and at each time step (corresponding to one RO) simulator uses the measured shift current, and gradient strength, and measured gantry angle to calculate the location of the FFL. The ideal simulated FFL is convolved with a 7 mm standard deviation Gaussian kernel. The 7 mm kernel width was chosen as an approximation of the imager’s native spatial resolution given the gradient strength and SPION’s saturation magnetization.

The matrix corresponding to this forward model is then inverted with MATLAB’s built-in preconditioned conjugate gradient solver, *pcg(...)* and uses 15 iterations, which is picked to balance image sharpness and artifact amplification. Fewer iterations yield smoother images, and more iterations result in sharper images with more ringing artifacts.

### 2.3. Imaging performance tests

#### 2.3.1. Spatial resolution

To evaluate the spatial resolution, we imaged two capillary tubes of 0.5 mg$ _{\mathrm{Fe}}\,\mathrm{mL}^{-1}$ Synomag. The tubes were 2.5 mm diameter, $\mathrm{\sim5\,cm}$ long and were spaced in 1 mm increments between 5 and 9 mm apart. A drive amplitude 5.8 mT$ _{\mathrm{peak}}$ was used for the image. For these test images, the shift currents were reduced by roughly 50% so the FFL moved only $\mathrm{\pm 37\,mm}$ to provide a 74 mm image FOV. This was done to zoom-in and increase the sampling density across the phantom. Note that the image resolution is primarily determined by the SPION’s magnetization curve and gradient strength. The speed of the FFL traversing the FOV (2.7 Hz) is well below the speed at which the relaxation times (on the order of microseconds (Mattingly *et al* 2024a)) would contribute to the spatial resolution.

The image datasets were reconstructed with both the inverse Radon and forward-model iterative algorithms. The images from both reconstructions were smoothed with a small 2D Gaussian kernel with a FWHM of 1.5 mm, which is well below the expected spatial resolution of the system based on the gradient strength and SPION properties, to suppress noise at higher spatial frequencies than our spatial resolution would permit.

To measure the spatial resolution, a line was plotted through the middle of the reconstructed images and the two most prominent peaks were identified in addition to the minimum signal intensity between the two peaks. The image contrast is defined as:

\begin{equation*} C = 1 - \frac{S_\textrm{min}}{0.5*\left(S_\textrm{max,Left}+S_\textrm{max,Right}\right)}\end{equation*}

where *C* is the dimensionless contrast metric, $S_\textrm{min}$ is the minimum signal between the adjacent peaks, and $S_\textrm{max,Left/Right}$ are the left and right peaks, respectively. A contrast of greater than 0.5 is considered resolved.

#### 2.3.2. Sensitivity

We measure the detection limit (sensitivity to iron) of the system using an 8-sample dilution series of Synomag ranging from undiluted (6 mg$ _{\mathrm{Fe}}\,\mathrm{mL}^{-1}$) to 15.6 *µ*g$ _{\mathrm{Fe}}$ ml^−1^. The initial concentration was taken from the manufacturer’s technical datasheet and the following were derived with a serial dilution. For each concentration, 20 *µ*L is pipetted in a small centrifuge tube to approximate a point-source phantom which is imaged in 5 s. The FOV for all images is 181 mm and the drive amplitude is 5.8 mT$ _{\mathrm{peak}}$. For the dilution series imaging, the phantom was carefully placed in a holder at a fixed position in the bore to ensure a repeatable placement. These data are reconstructed with both reconstruction algorithms and smoothed with a 6 mm FWHM Gaussian kernel to suppress noise at higher spatial frequencies than could be practically resolved. This kernel width is chosen based on the results of the spatial resolution tests. The image signal intensity was recorded from the location of peak signal in the most concentrated image and compared to the noise standard deviation of all voxels with the FOV of an empty-bore image. After linear fitting to determine the SNR as a function of iron mass, the iron mass expected to provide an SNR of 5 is defined as the detection limit.

#### 2.3.3. FOV measurement

The FOV is quantified with a large ‘G’-shaped phantom imaged with a shift current of 250 A$ _{\mathrm{peak}}$ to provide an expected FOV of 19 cm. The phantom was filled with 0.0625 mg$ _{\mathrm{Fe}}\,\mathrm{mL}^{-1}$ Synomag, which is roughly the concentration of blood assuming 5 mg$ _{\mathrm{Fe}}$ ml^−1^ dose and 65 ml of blood per kg body mass. The circular part of the ‘G’ is 136 mm in diameter, and that known distance is used to calibrate the total diameter of the FOV. It was important to have this calibration signal be well contained within the edges of the reconstruction, because toward the edge of the FOV the peak signal (indicating the center of the lines in the letter) may be more challenging to identify since the edges of the FOV are used as a baseline in post-processing.

#### 2.3.4. Thermal stability

To test the drive coils cooling ability over the course of a typical experiment, we monitored the drive current stability (and thus field stability) during 35 min of consecutive images with a drive field of 4.6 mT$ _{\mathrm{peak}}$ ($\mathrm{\sim}$40 A$ _{\mathrm{peak}}$). Since dissipated power scales with the square of the drive current, these results can be scaled to different drive currents (and thus drive fields). Upon completion of these images, we removed the drive coil from the bore and took off the shield and used a thermal camera to measure the temperature of the drive coils, capacitors, and surrounding structures.

## 3. Results

### 3.1. Spatial resolution

**Figure 7.**
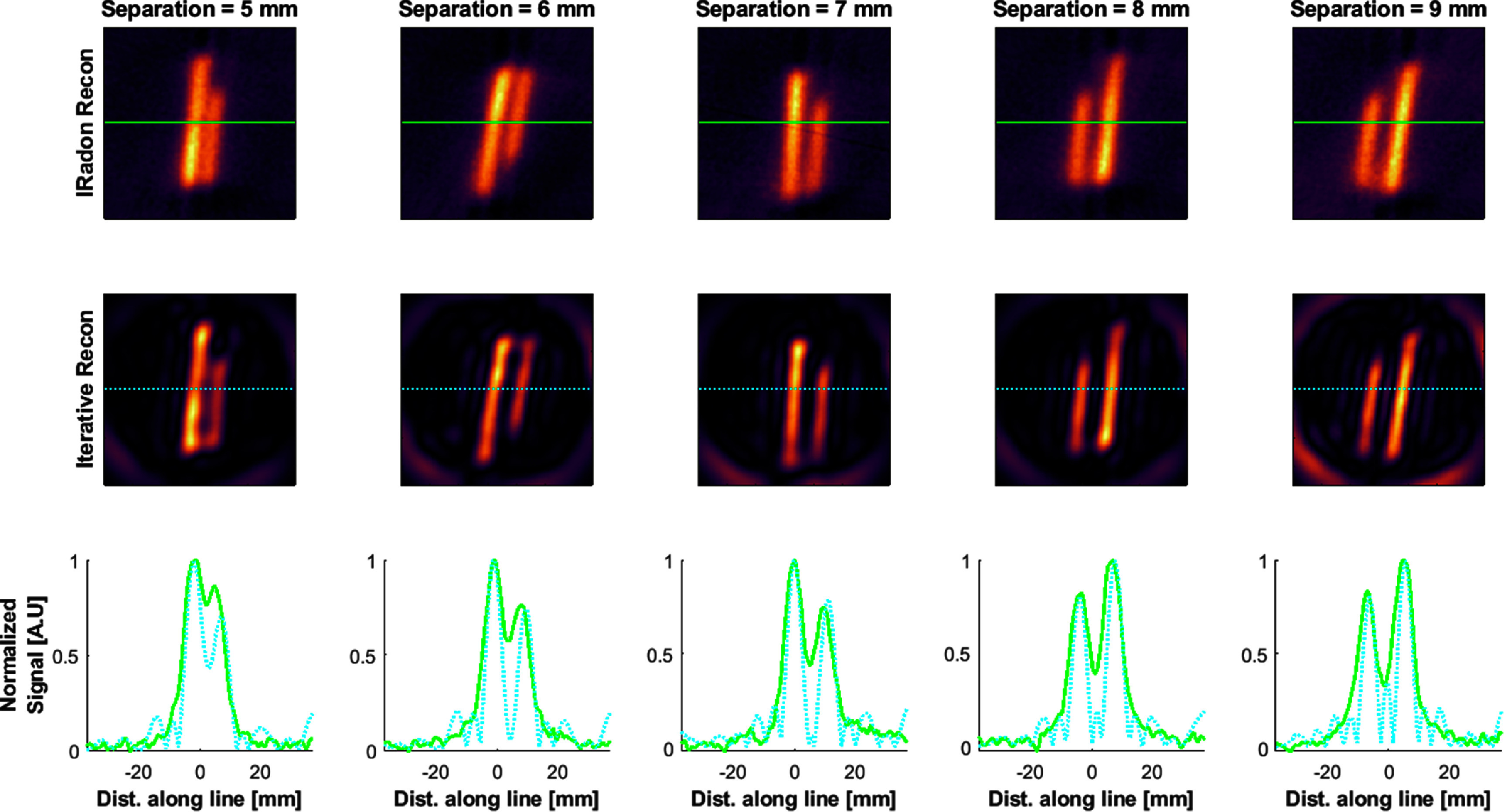
Reconstructions of spatial resolution phantoms. The field of view for each reconstruction is 74 mm diameter. The top row uses an inverse Radon reconstruction, and the middle row uses the iterative reconstruction. The bottom row contains plots of the normalized signal magnitude through the center of the image (green solid line = inv. Radon, cyan dashed = iterative recon).

**Figure 8.**
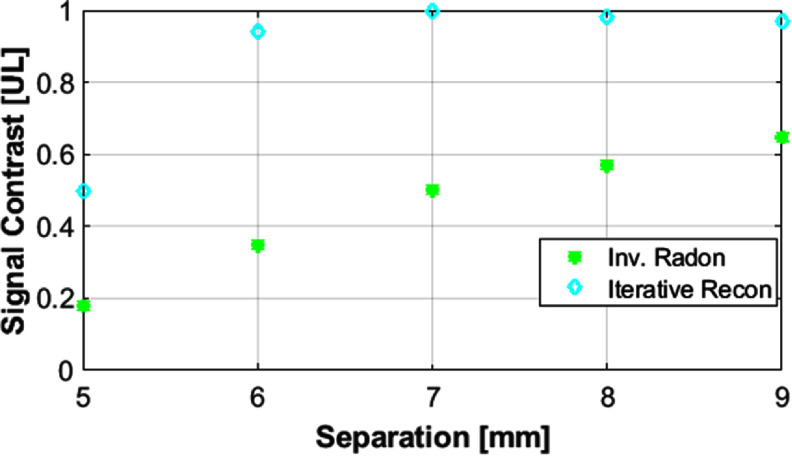
Image contrast (defined as one minus the mean of the peaks divided by the minimum signal between the peaks) for the reconstructions in figure 7.

Figures 7 and 8 show the reconstructions of the spatial resolution phantoms and the resulting signal contrast using the iterative reconstruction as well as the simple inverse Radon reconstruction. As expected, the iterative reconstruction considerably increases the spatial resolution to 5 mm, whereas the inverse Radon reconstruction cannot fully resolve the lines until about 7 mm distance between the inner surface lines. The iterative reconstruction introduces noticeable ringing artifacts while solving the inherently ill-posed deconvolution problem. The differences in the lengths of the lines are due to discrepancies in filling of the phantoms.

### 3.2. Imager sensitivity

**Figure 9.**
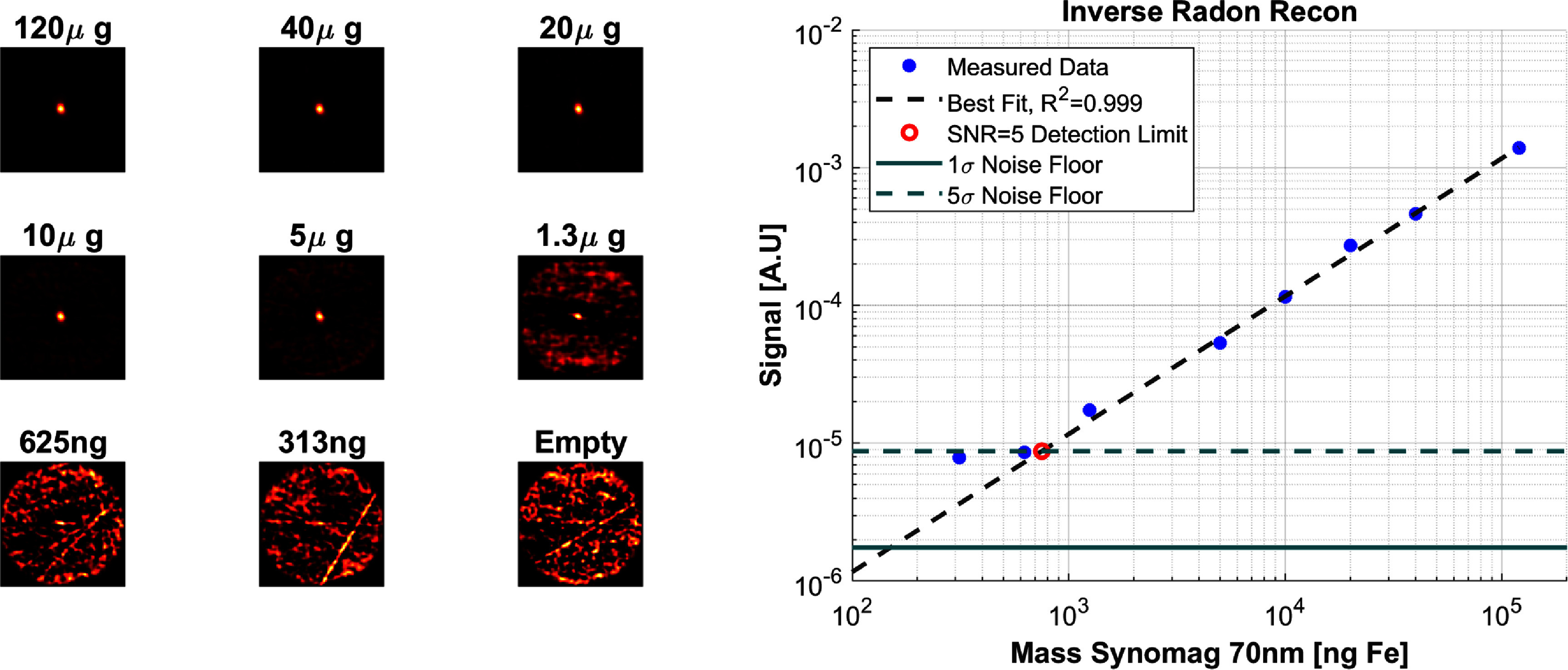
Left: Images of the dilution series (5 s each) ranging from 120 *µ*g$ _{\mathrm{Fe}}$ to 313 ng$ _{\mathrm{Fe}}$ and an empty bore image for comparison each scaled to the image maximum. Right: The linear regression for the signal versus SPION mass. The $\mathrm{1\sigma}$ and $\mathrm{5\sigma}$ noise floors are plotted as horizontal lines.

Figure 9 shows the imaging results from the Synomag dilution series using the inverse Radon reconstruction. The resulting images are linear, as expected, with an $\mathrm{\textit{R}^2 = 0.999}$, and only significantly diverge near the noise floor due to non-zero baseline signal. The best-fit line intercepts the empty-bore standard deviation (noise floor with $\mathrm{1\sigma}$) at a mass of 150 ng$ _{\mathrm{Fe}}$, and the $\mathrm{5\sigma}$ line at an Fe mass of 751 ng$ _{\mathrm{Fe}}$. The iterative reconstruction (data not shown) has similarly linear results with an $\mathrm{\textit{R}^2 = 0.999}$, but lower sensitivity as the deconvolution introduces some signal instability. The iterative reconstruction best-fit line intercepts the $\mathrm{1\sigma}$ horizontal line at a mass of 215 ng$ _{\mathrm{Fe}}$, and the $\mathrm{5\sigma}$ line at a Fe mass of 1077 ng$ _{\mathrm{Fe}}$.

### 3.3. FOV

**Figure 10.**
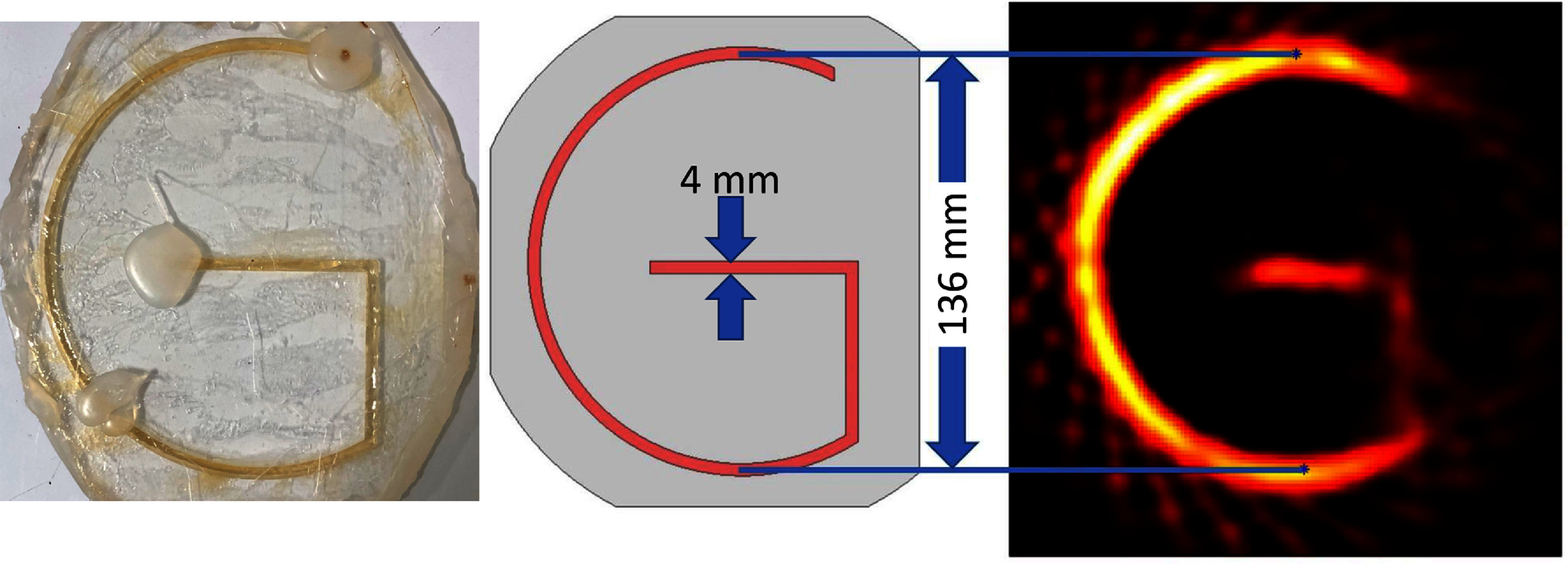
Left: Photograph of the ‘G’ phantom filled with 0.0625 mg Fe/ml of Synomag. Middle: rendering of the phantom with red illustrating where the Synomag is filling. The center diameter of the semi-circular component is 136 mm and the line width is 4 mm. Right: Inverse radon reconstruction of the data using 3 images (5 s each) averaged together.

Figure 10 illustrates the achievable FOV of the imager with a ‘G’ phantom extending 136 mm in diameter. By using this 136 mm known dimension, the entire imager FOV (distance between outermost pixels) is extrapolated to be 181 mm.

### 3.4. Thermal stability

**Figure 11.**
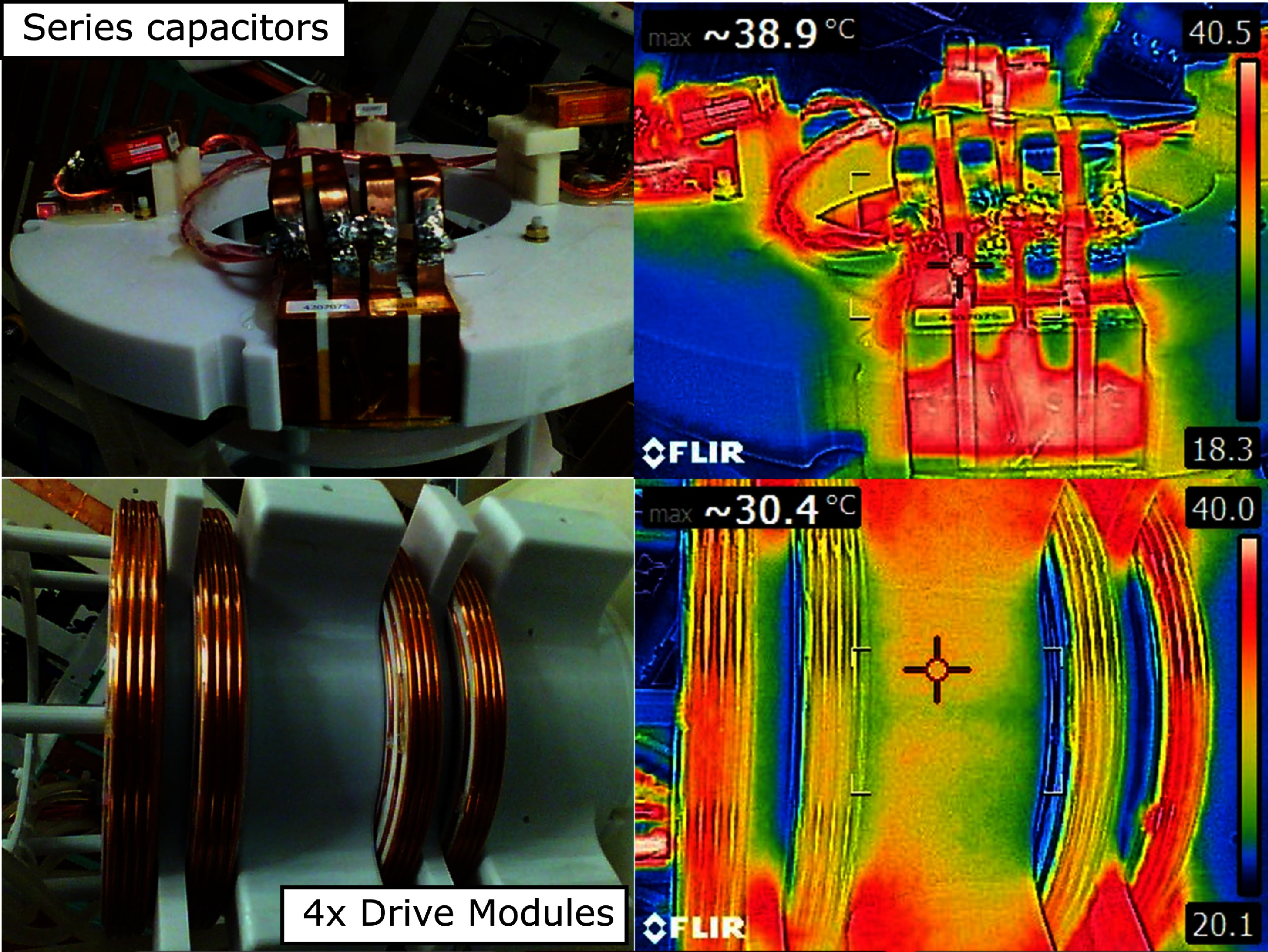
Temperatures as measured following a $\mathrm{\sim}$35 min experiment at 4.5 mT$ _{\mathrm{peak}}$. Top: Capacitors in series with the drive coil. Bottom: Drive coil wires and former.

Figure 11 shows the thermal images and the corresponding visible-light images following $\mathrm{\sim}$35 min of continuous imaging. Neither the drive coil nor the capacitors exceeded approximately $\mathrm{40^{\circ}C}$. Notably, the outer components of the 3D printed structure (white plastic components) are hotter than the drive coil itself due to their proximity to the copper shield, which is being heated via eddy currents. The amplitude of the current in the drive coil deviated less than 2% during the 35 min continuous imaging test.

## 4. Discussion

We present the first human-head-sized mechanically rotating FFL-based MPI system. It achieves a spatial resolution, a temporal resolution (2D image), and a sensitivity that is appropriate for functional human neuroimaging. It employs the highest localizing gradient to date (1.13 T m^−1^ in *x*’) for an MPI system with a FOV suitable for adult human heads (Lee *et al* 2006, Rahmer *et al* 2018, Graeser *et al* 2019). In addition to potentially enabling future clinical studies on this prototype instrument, it demonstrates the ability to scale a number of different technical MPI approaches to human sized imagers, including the use of FFL assemblies based on continuously rotating permanent magnets and shift coils and high sensitivity gradiometer based receive coils. By employing other recent developments in human-scale MPI, such as optimized drive coil wire, mitigating connection-induced distortions, using optimized FFL scanning trajectories, among others (Top *et al* 2019, Kretov *et al* 2024, Mohn *et al* 2024, Ozaslan *et al* 2024, Trisnanto *et al* 2024, Mattingly *et al* 2024c), the system’s performance could be significantly improved.

### 4.1. Spatial resolution

We demonstrated spatial resolution of approximately 5 mm with an iterative reconstruction and 7 mm with an inverse Radon reconstruction that does not try to deconvolve the SPION’s point-spread function. Image resolution is not determined by hardware parameters (such as FFL gradient strength) alone, but is also a strong function of the SPION used (Bauer *et al* 2016, Wang *et al* 2020, Tay *et al* 2021, Fung *et al* 2023, Abdibastami *et al* 2024), drive field amplitude and frequency (Croft *et al* 2016, Tay *et al* 2017, 2019b), and reconstruction deconvolution effort. The iterative reconstruction we demonstrate here could be significantly improved by utilizing many of the recent developments in MPI reconstruction algorithms (Knopp *et al* 2019, Chacon-Caldera *et al* 2020, 2021, Gungor *et al* 2023, Sanders *et al* 2024). The scanner’s demonstrated 5 mm spatial resolution and 5 s temporal resolution was designed for detecting CBV increases associated with functional brain activation and is comparable to that used in many MRI based functional neuroimaging applications. While MRI can flexibly achieve higher spatial and temporal resolutions, the majority of published studies are acquired with a temporal resolution of a few seconds and apply a spatial smoothing filter ranging from 4–12 mm to the acquired image (Sacchet and Knutson 2013, Triana *et al* 2020). Our MPI scanner’s demonstrated spatial resolution would also likely be sufficient for observing the CBV alterations associated with human traumatic brain injuries.

Applications requiring increased spatial resolution will likely require improvement in the SPION’s magnetization response or utilization of superconducting FFL and shift coil magnets. Recent developments in SPION chemistry suggest up to 10 times higher spatial resolution would be possible (Tay *et al* 2021) and superconducting magnets are being developed for MPI at large scales with up to $2.5~\mathrm{Tm}^{-1}$ gradient (Le *et al* 2024) which would increase the spatial resolution another factor of 2.5 from what we have demonstrated. Future work includes further characterization of the modulation transfer function with varying acquisition parameters and testing with standard resolution phantoms (e.g. a Derenzo phantom (Cox *et al* 2016)) to facilitate comparison to other modalities.

### 4.2. Imager sensitivity

The Synomag dilution series images demonstrate a SNR = 5 (best fit intercept) with 751 ng$ _{\mathrm{Fe}}$ with an inverse Radon reconstruction and 1077 ng$ _{\mathrm{Fe}}$ with the iterative reconstruction. Thus the practical detection limit, approximating with one significant figure, is about 1 *µ*g$ _{\mathrm{Fe}}$ for either reconstruction (5 section image, 5.8 mT$ _{\mathrm{peak}}$, 6 mm FWHM smoothing kernel) because of the non-Gaussian nature of the background signal. This limit of detection (LOD) should be thought of as a point of reference rather than a fixed parameter of the imager. It is a strong function of scan time ($\mathrm{LOD\propto \sqrt{1/time}}$), drive field amplitude (LOD improves with higher drive fields), amount of smoothing (LOD improves with more smoothing), SPION used, etc.

Based on the drug labeling for Ferumoxytol (Adkinson *et al* 2018)), human SPION doses can be expected to contain 5 mg Fe per kg body mass. Assuming 65 ml blood per kg body mass, the blood concentration of Fe will be 78 *µ*g$ _{\mathrm{Fe}}$ ml^−1^. The brain’s gray matter is about 5% blood, so the mass of Fe per tissue volume in gray matter is expected to be 3.8 *µ*g$ _{\mathrm{Fe}}$ ml^−1^. The imager produces 6 mm isotropic voxels, or 216 *µ*L, yielding an Fe mass per voxel of 830 ng$ _{\mathrm{Fe}}$ in cerebral gray matter. For these values, the inverse Radon reconstruction images should show gray matter with an SNR of about 5 and large blood vessels with an SNR of about 100 in the 5 s image. A functional task, such as flashing lights, is expected to increase CBV by 25% (Mandeville *et al* 1998) and hypercapnia challenges in rodents have induced similar MPI detected blood volume changes (Mason *et al* 2023). Based on our sensitivity results, we expect to see this 25% CBV change with a contrast-to-noise ratio (CNR) above 1 in the 5 section image.

Currently, the scanner’s sensitivity is limited by an unstable background signal originating in the shift coil amplifier and then coupled into the receive system. Noise measurements with and without the shift system energized show that this noise source is approximately 10x higher than the receive preamplifier’s noise floor, motivating future work to mitigate this source with improved filtering of the shift coil currents as they pass into the shielded room. Similar noise from the drive coil amplifier raises the noise floor by another factor of two above the preamplifier noise floor. This motivates improving connections and filtering in the drive chain as well and reducing the pass-band width of the transmit filter (Biwa *et al* 2006, Krüger *et al* 2010, Wilkerson 2010).

With these fixes providing a roughly 20x sensitivity boost, we would expect to detect functional CBV changes during human brain activation with a CNR of roughly 20, exceeding the state of the art achieved by fMRI. This still does not represent the body-noise dominated case whereby the receive chain noise level is set by dissipative losses in the body (rather than the preamp or receive coils themselves). To achieve body noise domination, the noise from the preamplifier and receive coil losses must be addressed. The preamplifier’s noise floor could be reduced with a number of designs suggested for MPI-tailored preamps. For example, a voltage noise at or below 150 pV/$\sqrt{\mathrm{Hz}}$ has been achieved by using low-noise JFETs such as the 2SK2394(On Semi, Scottsdale, Arizona) or BF862(NXP, Eindhoven, Netherlands) in a parallelized common-source configuration (Schmale *et al* 2010, Graeser *et al* 2017, Mattingly *et al* 2022a). Similarly low noise values have also been achieved with transformer matching or parallel opamp inputs (Zheng *et al* 2017, Malhotra *et al* 2020). Due to the large inductance of the receive coils, cascoding the input JFETs (Sedra and Smith 2004, Huynh 2018) or using other hybrid amplifier designs (Horowitz and Hill 2015) may be necessary to mitigate the Miller effect capacitance (Sedra and Smith 2004), but this comes at the expense of complexity. These suggest roughly another order of magnitude lower noise floor could be available. For MRI-based functional neuroimaging, the noise in the image timeseries is dominated by the ‘physiological noise,’ which originates from numerous biological functions uncorrelated to the given task under investigation (Triantafyllou *et al* 2006). These biological processes impose nuisance fluctuations on the large signal baseline, only a small fraction of which derives from the blood pool. Because MPI detects only the cerebral blood pool and therefore eliminates much of the signal susceptible to modulation, it is likely less susceptible to these sources of instability (e.g. respiration artifacts from the magnetization of the air in the lungs, spin history effects, etc). Thus, the biological signal fluctuations would be primarily CBV fluctuations, which are the source of valuable functional connectivity information.

### 4.3. FOV

The FOV shown in figure 10 is sufficient to cover the cross-section of most human brains. This is partially due to the shift amplifiers which require repair to operate at their specified power, and limits the FOV to about 83% of the system design. With this repair, the expected FOV is 220 mm, and should comfortably encompass the head. The imaging reconstructions are also 2D in their current formulation, but this is not an inherent limitation of the device. To encode a third dimension (the *z* axis), there are a few possible approaches: the subject could be moved in an automated manner, as done in spiral CT, or as in pre-clinical mechanically rotating FFL devices (Mattingly *et al* 2022a), a DC-offset current could be added to the drive coil, or another low-frequency coil could be added to shift the FFL in the *z* axis. Any approach to encoding in the third dimension would have to consider the effects on temporal resolution for each frame of an fMPI timeseries. This would additionally require improvements to the reconstruction to include 3D information in the model, which has been done with the SLICE reconstruction on the similar architecture rodent-scale MPI system (Mason *et al* 2022), or other 3D reconstruction algorithms (e.g. system matrix (Graeser *et al* 2019) or *X*-Space (Goodwill and Conolly 2011)).

### 4.4. Temporal stability

**Figure 12.**
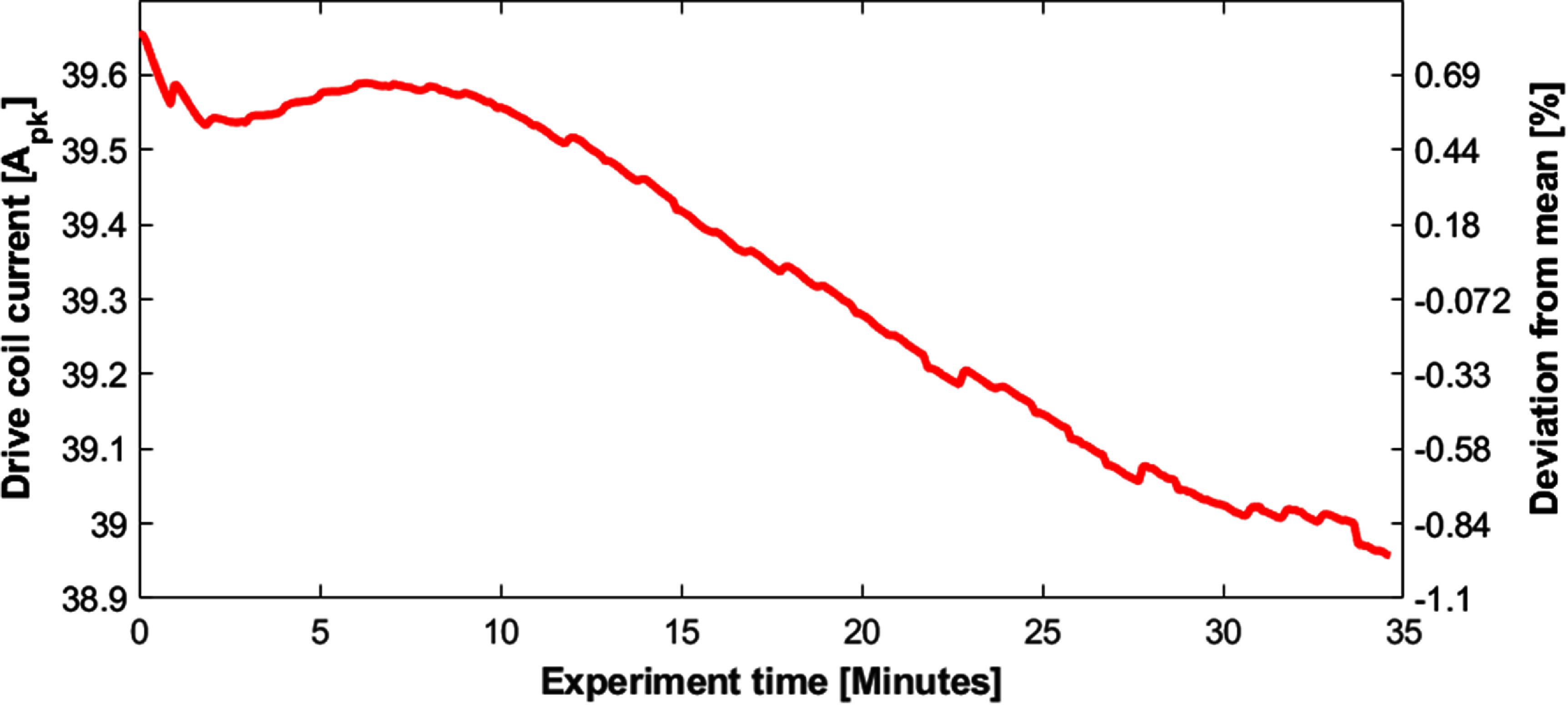
Drive coil current over the course of a $\mathrm{\sim}$35 min continuous imaging experiment.

For time-series imaging, having thermal stability is critical. Figures 11 and 12 show the temperature and drive coil currents after over 30 min of continuous imaging. Over the course of the experiment, the coil current drifted less than 2%. The drift was generally smooth, slow, and relatively low amplitude compared to the $\mathrm{\sim25\%}$ changes expected from brain activation. Because of these features, it is straightforward to separate it out from the biological signal changes associated with brain activation using a linear model (Mason *et al* 2023). Altogether, it is unlikely thermal drift will be a prohibitive component in functional imaging experiments.

While the heating experiments were carried out with 40 A$ _{\mathrm{peak}}$, the temperatures we observed would scale roughly with the current squared. So, for 50 A$ _{\mathrm{peak}}$, the temperature rises would be about $\mathrm{(50/40)^2\times}$ or 50% higher, which would still be well below the threshold for damaging components. If needed, active cooling of the capacitors and the shielding tube could potentially be applied. Additionally, water coolant pressure could be increased if needed.

Another source of temporal drift is the washout of the SPIONs from circulation. In prior experiments using Synomag-D the half-life was measured as 48 min (Mason *et al* 2023), which is consistent with others’ findings (Szwargulski *et al* 2020, Liu *et al* 2021). Longer circulations are also possible by tuning the size and surface properties of the SPIONs. To account for this drift in the analysis, an additional exponential decay term can be included because the trend is slow and predictable.

### 4.5. Biological effects of the applied d*B*/d*t*

Human MPI will impose a number of different time-varying magnetic fields on the body that may induce peripheral nerve stimulation (PNS), tissue heating from the specific absorption rate (SAR), and magneto-stimulation of the retinas. The shift fields induce a magnetic field approximately 100 mT$ _{\mathrm{peak}}$ over the head at 2.7 Hz. Due to the low frequency of this applied field, the d*B*/d*t* and thus the induced electric fields in the body are relatively small. The FFL being rotated also causes a d*B*/d*t* but at even lower frequencies. While there is limited data on the topic, our prior analysis shows that for frequencies below 10 Hz stimulation is unlikely (Mason 2020) although retinal stimulation becomes a concern for frequencies of 20 Hz (Lovsund *et al* 1980).

For our drive field operating at 26.3 kHz at up to 7 mT$ _{\mathrm{peak}}$, Saritas *et al* have shown SAR is unlikely to be a limiting factor (Saritas *et al* 2013). The PNS limits in the head are still unclear, but preliminary studies have shown that the 7 mT$ _{\mathrm{peak}}$ may be at or near the limit (Ozaslan *et al* 2022, Barksdale *et al* 2024), and future work is needed to elucidate the safe limits.

## 5. Conclusions

We demonstrate, for the first time, that a human-scale MPI system with a sufficiently strong gradient ($1\,\mathrm{Tm}^{-1}$) to produce clinically-relevant images can be realized with high sensitivity (1 *µ*g$ _{\mathrm{Fe}}$) for continuous 5 s CBV imaging. We expect this device to be sufficient for seeing the hemodynamic modulations following brain activity *in vivo* although the instrument is still far from the expected limits of MPI detection sensitivity and further mitigation of noise sources will be needed to achieve the full potential of functional MPI. But, with the appropriate investment in engineering, human brain MPI can potentially provide an order of magnitude improvement in sensitivity at a useful spatial resolution bringing a new and high-sensitivity modality to human neuroimaging.

## Data Availability

The data that support the findings of this study are openly available at the following URL/DOI: https://github.com/EliMattingly22/Data_DesignConstrVal_HumanMPIManuscript.

## References

[pmbad9db0bib1] Abdibastami A (2024). How size and composition of cobalt doped iron oxide nanoparticle tracers enhance magnetic particle imaging performance. Chem. Mater..

[pmbad9db0bib2] Aderhold E, Graeser M, Wieprecht H (2023). Modular MPI component testing facility. Int. J. Magn. Part. Imaging.

[pmbad9db0bib3] Adkinson N F, Strauss W E, Macdougall I C, Bernard K E, Auerbach M, Kaper R F, Chertow G M, Krop J S (2018). Comparative safety of intravenous ferumoxytol versus ferric carboxymaltose in iron deficiency anemia: a randomized trial. Am. J. Hematol..

[pmbad9db0bib4] Barksdale A, Ferris N, Mattingly E, Sliwiak M, Guerin B, Wald L, Davids M, Klein V (2024). Measured PNS thresholds in a human head MPI solenoid from 200 Hz to 88.1 kHz. Int. J. Magn. Part. Imaging.

[pmbad9db0bib5] Bauer L M, Situ S F, Griswold M A, Samia A C S (2016). High-performance iron oxide nanoparticles for magnetic particle imaging-guided hyperthermia (hMPI). Nanoscale.

[pmbad9db0bib6] Biwa S, Hiraiwa S, Matsumoto E (2006). Experimental and theoretical study of harmonic generation at contacting interface. Ultrasonics.

[pmbad9db0bib7] Chacon-Caldera J, Lehr H, Sajjamark K, Franke J (2020). Eigen-reconstructions: a closer look into the system matrix. Int. J. Magn. Part. Imaging.

[pmbad9db0bib8] Chacon-Caldera J, Lehr H, Sajjamark K, Franke J (2021). Enhancing spatial resolution in magnetic particle imaging using eigen-reconstructions: opportunities and limitations. Int. J. Magn. Part. Imaging.

[pmbad9db0bib9] Chacon-Caldera J, Mattingly E, Sliwiak M, Barksdale A C, Niebel F H, Wald L L (2024). A receive insert for non-human primate functional MPI (fMPI). Int. J. Magn. Part. Imaging.

[pmbad9db0bib10] Cox B L, Graves S A, Farhoud M, Barnhart T E, Jeffery J J, Eliceiri K W, Nickles R J (2016). Development of a novel linearly-filled Derenzo microPET phantom. Am. J. Nucl. Med. Mol. Imaging.

[pmbad9db0bib11] Croft L R, Goodwill P W, Konkle J J, Arami H, Price D A, Li A X, Saritas E U, Conolly S M (2016). Low drive field amplitude for improved image resolution in magnetic particle imaging. Med. Phys..

[pmbad9db0bib12] DeYoe E A, Bandettini P, Neitz J, Miller D, Winans P (1994). Functional magnetic resonance imaging (FMRI) of the human brain. J. Neurosci. Methods.

[pmbad9db0bib13] Erbe M, Knopp T, Sattel T F, Biederer S, Buzug T M (2011). Experimental generation of an arbitrarily rotated field-free line for the use in magnetic particle imaging. Med. Phys..

[pmbad9db0bib14] Ergor M, Bingolbali A (2022). Field-free line magnetic particle imaging magnet design using nested halbach cylinders. IEEE Magn. Lett..

[pmbad9db0bib15] Ferkovic L, Ilic D (2007). Dependence of mutual inductance of a precise Rogowski coil on the primary conductor position.

[pmbad9db0bib16] Fung K L B (2023). First superferromagnetic remanence characterization and scan optimization for super-resolution magnetic particle imaging. Nano Lett..

[pmbad9db0bib17] Gleich B, Weizenecker J (2005). Tomographic imaging using the nonlinear response of magnetic particles. Nature.

[pmbad9db0bib18] Goodwill P W, Conolly S M (2010). The X-space formulation of the magnetic particle imaging process: 1-D signal, resolution, bandwidth, SNR, SAR and magnetostimulation. IEEE Trans. Med. Imaging.

[pmbad9db0bib19] Goodwill P W, Conolly S M (2011). Multidimensional X-space magnetic particle imaging. IEEE Trans. Med. Imaging.

[pmbad9db0bib20] Graeser M (2017). Towards picogram detection of superparamagnetic iron-oxide particles using a gradiometric receive coil. Sci. Rep..

[pmbad9db0bib21] Graeser M (2019). Human-sized magnetic particle imaging for brain applications. Nat. Commun..

[pmbad9db0bib22] Graeser M, Liebing T, Szwargulski P, Förger F, Thieben F, Ludewig P, Knopp T (2020). Organ specific mouse head coil for improved image quality in magnetic particle imaging. Int. J. Magn. Part. Imaging.

[pmbad9db0bib23] Greiner C, Rückert M A, Kampf T, Behr V C, Vogel P (2022). Traveling wave MPI utilizing a field-free line. Int. J. Magn. Part. Imaging.

[pmbad9db0bib24] Gungor A, Askin B, Soydan D A, Saritas E U, Top C B, Cukur T (2023). A denoiser scaling technique for plug-and-play MPI reconstruction. Int. J. Magn. Part. Imaging.

[pmbad9db0bib25] Horowitz P, Hill W (2015). The Art of Electronics.

[pmbad9db0bib26] Huettel S A, Song A W, McCarthy a G (2014). Functional Magnetic Resonance Imaging.

[pmbad9db0bib27] Huynh Q (2018). Ultra low noise preamplifier design for magnetic particle imaging. Master’s Thesis.

[pmbad9db0bib28] Khandhar P, Keselman A, Kemp P J, Ferguson S M, Goodwill R W, Conolly P M, Krishnan M (2017). Evaluation of PEG-coated iron oxide nanoparticles as blood pool tracers for preclinical magnetic particle imaging. Nanoscale.

[pmbad9db0bib29] Kluth T (2018). Mathematical models for magnetic particle imaging. Inverse Problems.

[pmbad9db0bib30] Knopp T, Gdaniec N, Rehr R, Graeser M, Gerkmann T (2019). Correction of linear system drifts in magnetic particle imaging. Phys. Med. Biol..

[pmbad9db0bib31] Knopp T, Sattel T F, Biederer S, Buzug T M (2010a). Field-free line formation in a magnetic field. J. Phys. A: Math. Theor..

[pmbad9db0bib32] Knopp T, Sattel T F, Biederer S, Rahmer J, Weizenecker J, Gleich B, Borgert J, Buzug T M (2010b). Model-based reconstruction for magnetic particle imaging. IEEE Trans. Med. Imaging.

[pmbad9db0bib33] Kretov E, Scheel J-P, Mirzojan L, Sevecke F, Graeser M (2024). Power-optimized drive field coils for human brain magnetic particle imaging. Int. J. Magn. Part. Imaging.

[pmbad9db0bib34] Krüger M, Nils F N, Reichl H (2010). Intermodulation distortion as indicator for interconnect degradation.

[pmbad9db0bib35] Le T-A, Bui M P, Hadadian Y, Gadelmowla K M, Oh S, Im C, Hahn S, Yoon J (2024). Towards human-scale magnetic particle imaging: development of the first system with superconductor-based selection coils.

[pmbad9db0bib36] Lee J-h, Shin S-j H, Istook C L (2006). Analysis of human head shapes in the United States. Int. J. Human Ecol..

[pmbad9db0bib37] Liu S, Chiu-Lam A, Rivera-Rodriguez A, DeGroff R, Savliwala S, Sarna N, Rinaldi-Ramos C M (2021). Long circulating tracer tailored for magnetic particle imaging. Nanotheranostics.

[pmbad9db0bib38] Lovsund P, Oberg P, Nilsson S E G (1980). Magneto- and electrophosphenes: a comparative study. Med. Biol. Eng. Comput..

[pmbad9db0bib39] Ludewig P (2017). Magnetic particle imaging for real-time perfusion imaging in acute stroke. ACS Nano.

[pmbad9db0bib40] Malhotra A, Schwegmann H, Schumacher J, Chen X, Buzug T M (2020). Fully differential low noise amplifier for MPI/MPS. Int. J. Magn. Part. Imaging.

[pmbad9db0bib41] Mandeville J B, Marota J J, Kosofsky B E, Keltner J R, Weissleder R, Rosen B R, Weisskoff R M (1998). Dynamic functional imaging of relative cerebral blood volume during rat forepaw stimulation. Magn. Reson. Med..

[pmbad9db0bib42] Mason E E (2020). Magnetic particle imaging technology for clinical intraoperative breast cancer margin assessment and functional brain imaging. PhD Thesis.

[pmbad9db0bib43] Mason E E (2023). Functional magnetic particle imaging (fMPI) of cerebrovascular changes in the rat brain during hypercapnia. Phys. Med. Biol..

[pmbad9db0bib44] Mason E E (2024). Preliminary results: large bore clinical MPI system imaging human head-sized FOVs. Int. J. Magn. Part. Imaging.

[pmbad9db0bib45] Mason E E, Cauley S F, Mattingly E, Sliwiak M, Wald L L (2022). Side lobe informed center extraction (SLICE): a projection-space forward model reconstruction for a 2D imaging system. Int. J. Magn. Part. Imaging.

[pmbad9db0bib46] Mason E E, Cooley C Z, Cauley S F, Griswold M A, Conolly S M, Wald L L (2017). Design analysis of an MPI human functional brain scanner. Int. J. Magn. Part. Imaging.

[pmbad9db0bib47] Mattingly E (2024). Design, construction, and validation of magnetic particle imaging systems for rodent, primate, and human functional neuroimaging.. Thesis.

[pmbad9db0bib48] Mattingly E, Barksdale A C, Sliwiak M, Chacon-Caldera J, Mason E E, Wald L L (2024a). Open-source device for high sensitivity magnetic particle spectroscopy, relaxometry and hysteresis loop tracing. Rev. Sci. Instrum..

[pmbad9db0bib49] Mattingly E, Mason E E, Herb K, Sliwiak M, Drago J, Graeser M, Wald L L (2022a). A sensitive, stable, continuously rotating FFL MPI system for functional imaging of the rat brain. Int. J. Magn. Part. Imaging.

[pmbad9db0bib50] Mattingly E, Sliwiak M, Chacon-Caldera J, Barksdale A, Niebel F, Mason E, Wald L (2024b). A human-scale magnetic particle imaging system for functional neuroimaging. Int. J. Magn. Part. Imaging.

[pmbad9db0bib51] Mattingly E, Sliwiak M, Chacon-Caldera J, Barksdale A, Wald L (2024). Optimization of wall thickness for water-cooled hollow conductor drive coils in human-sized MPI. Int. J. Magn. Part. Imaging.

[pmbad9db0bib52] Mattingly E, Sliwiak M, Drago J M, Mason E E, Graeser M, Wald L L (2022b). A drive filter design for MPI with harmonic notching and selective damping. Int. J. Magn. Part. Imaging.

[pmbad9db0bib53] Mohn F, Forger F, Thieben F, Moddel M, Schmale I, Knopp T, Graeser M (2024). Resonant inductive coupling network for human-sized magnetic particle imaging. Rev. Sci. Instrum..

[pmbad9db0bib54] Nomura K, Washino M, Matsuda T, Seino S, Nakagawa T, Kiwa T, Kanemaru M (2024). Development of human head size magnetic particle imaging system. Int. J. Magn. Part. Imaging.

[pmbad9db0bib55] Orendorff R (2017). First *in vivo* traumatic brain injury imaging via magnetic particle imaging. Phys. Med. Biol..

[pmbad9db0bib56] Ozaslan A A, Babaloo R, Atalar E, Saritas E U (2024). Minimizing induced electric fields in human head-size MPI Systems. Int. J. Magn. Part. Imaging.

[pmbad9db0bib57] Ozaslan A A, Utkur M, Canpolat U, Tuncer M A, Oguz K K, Saritas E U (2022). PNS limits for human head-size MPI systems: preliminary results. Int. J. Magn. Part. Imaging.

[pmbad9db0bib58] Pagan J, McDonough C, Vo T, Tonyushkin A (2021). Single-sided magnetic particle imaging device with field-free-line geometry for *in vivo* imaging applications. IEEE Trans. Magn..

[pmbad9db0bib59] Rahmer J, Stehning C, Gleich B (2018). Remote magnetic actuation using a clinical scale system. PLoS One.

[pmbad9db0bib60] Rahmer J, Weizenecker J, Gleich B, Borgert J (2009). Signal encoding in magnetic particle imaging: properties of the system function. BMC Med. Imaging.

[pmbad9db0bib61] Ramboz J (1996). Machinable Rogowski coil, design and calibration. IEEE Trans. Instrum. Meas..

[pmbad9db0bib62] Sacchet M D, Knutson B (2013). Spatial smoothing systematically biases the localization of reward-related brain activity. NeuroImage.

[pmbad9db0bib63] Sanders T, Mason E, Konkle J, Goodwill P (2024). Multi-harmonic gridded 3D deconvolution (MH3D) for image reconstruction in MPI: MH3D. Int. J. Magn. Part. Imaging.

[pmbad9db0bib64] Saritas E U, Goodwill P W, Zhang G Z, Conolly S M (2013). Magnetostimulation limits in magnetic particle imaging. IEEE Trans. Med. Imaging.

[pmbad9db0bib65] Sattel T F, Knopp T, Biederer S, Gleich B, Weizenecker J, Borgert J, Buzug T M (2009). Single-sided device for magnetic particle imaging. J. Phys. D: Appl. Phys..

[pmbad9db0bib66] Schmale I, Gleich B, Borgert J, Weizenecker J (2010). Jfet noise modelling for MPI receivers. Magn. Nanopart..

[pmbad9db0bib67] Schumacher J, Malhotra A, Grafe K, Buzug T M (2020). Highly symmetric filter for a fully differential receive chain. Int. J. Magn. Part. Imaging.

[pmbad9db0bib68] Sedra A S, Smith K C (2004). Microelectronic Circuits (The Oxford Series in Electrical and Computer Engineering ).

[pmbad9db0bib69] Shliomis M (1972). Effective viscosity of magnetic fluids. J. Exp. Theor. Phys..

[pmbad9db0bib70] Szwargulski S (2020). Monitoring intracranial cerebral hemorrhage using multicontrast real-time magnetic particle imaging. ACS Nano.

[pmbad9db0bib71] Tay Z W (2021). Superferromagnetic nanoparticles enable order-of-magnitude resolution & sensitivity gain in magnetic particle imaging. Small Methods.

[pmbad9db0bib72] Tay Z W, Hensley D W, Chandrasekharan P, Zheng B, Conolly S M (2019b). Optimization of drive parameters for resolution, sensitivity and safety in magnetic particle imaging. IEEE Trans. Med. Imaging.

[pmbad9db0bib73] Tay Z W, Hensley D W, Vreeland E C, Zheng B, Conolly S M (2017). The relaxation wall: experimental limits to improving MPI spatial resolution by increasing nanoparticle core size. Biomed. Phys. Eng. Express.

[pmbad9db0bib74] Tay Z W, Hensley D, Ma J, Chandrasekharan P, Zheng B, Goodwill P, Conolly S (2019a). Pulsed excitation in magnetic particle imaging. IEEE Trans. Med. Imaging.

[pmbad9db0bib75] Thieben F, Foerger F, Mohn F, Hackelberg N, Boberg M, Scheel J-P, Moddel M, Graeser M, Knopp T (2024). System characterization of a human-sized 3D real-time magnetic particle imaging scanner for cerebral applications. Commun. Eng..

[pmbad9db0bib76] Top C B D, Ilbey S, Guven H E (2017). Electronically rotated and translated field-free line generation for open bore magnetic particle imaging:. Med. Phys..

[pmbad9db0bib77] Top C B, Gungor A, Ilbey S, Guven H E (2019). Trajectory analysis for field free line magnetic particle imaging. Med. Phys..

[pmbad9db0bib78] Triana A M, Glerean E, Saramaki J, Korhonen O (2020). Effects of spatial smoothing on group-level differences in functional brain networks. Netw. Neurosci..

[pmbad9db0bib79] Triantafyllou C, Hoge R D, Wald L L (2006). Effect of spatial smoothing on physiological noise in high-resolution fMRI. NeuroImage.

[pmbad9db0bib80] Trisnanto S B, Kasajima T, Shibuya T, Takemura Y (2024). Gradiometric human head coil-coupled magnetoresistive sensor for sensitivity improvement in magnetic particle imaging. Int. J. Magn. Part. Imaging.

[pmbad9db0bib81] Vogel P, Ruckert M A, Greiner C, Gunther J, Reichl T, Kampf T, Bley T A, Behr V C, Herz S (2023). iMPI: portable human-sized magnetic particle imaging scanner for real-time endovascular interventions. Sci. Rep..

[pmbad9db0bib82] Wang Q, Ma X, Liao H, Liang Z, Li F, Tian J, Ling D (2020). Artificially engineered cubic iron oxide nanoparticle as a high-performance magnetic particle imaging tracer for stem cell tracking. ACS Nano.

[pmbad9db0bib83] Weber M, Beuke J, Gladiss A v, Grafe K, Vogel P, Behr V C, Buzug T M (2018). Novel field geometry using two halbach cylinders for FFL-MPI. Int. J. Magn. Part. Imaging.

[pmbad9db0bib84] Weizenecker J, Gleich B, Borgert J (2008). Magnetic particle imaging using a field free line. J. Phys. D: Appl. Phys..

[pmbad9db0bib85] Weizenecker J, Gleich B, Rahmer J, Dahnke H, Borgert J (2009). Three-dimensional real-time *in vivo* magnetic particle imaging. Phys. Med. Biol..

[pmbad9db0bib86] Wilkerson J R (2010). Passive intermodulation distortion in radio frequency communication systems. PhD Thesis.

[pmbad9db0bib87] Wu L (2019). A review of magnetic particle imaging and perspectives on neuroimaging. Am. J. Neuroradiol..

[pmbad9db0bib88] Yoshida T, Kamei Y, Takemura Y, Nagano T, Ide A, Sasayama T (2024). Development of magnetic particle imaging modules using high-Tc superconducting coils. Int. J. Magn. Part. Imaging.

[pmbad9db0bib89] Yu E Y (2017). Magnetic particle imaging for highly sensitive, quantitative and safe *in vivo* gut bleed detection in a murine model. ACS Nano.

[pmbad9db0bib90] Zheng B, Goodwill P W, Dixit N, Xiao D, Zhang W, Gunel B, Lu K, Scott G C, Conolly S M (2017). Optimal broadband noise matching to inductive sensors: application to magnetic particle imaging. IEEE Trans. Biomed. Cir. Syst..

[pmbad9db0bib91] Zigrang D J, Sylvester N D (1985). A review of explicit friction factor equations. J. Energy Resour. Technol. Trans. ASME.

